# Lysine methylation promotes NFAT5 activation and determines temozolomide efficacy in glioblastoma

**DOI:** 10.1038/s41467-023-39845-z

**Published:** 2023-07-10

**Authors:** Yatian Li, Zhenyue Gao, Yuhong Wang, Bo Pang, Binbin Zhang, Ruxin Hu, Yuqing Wang, Chao Liu, Xuebin Zhang, Jingxuan Yang, Mei Mei, Yongzhi Wang, Xuan Zhou, Min Li, Yu Ren

**Affiliations:** 1 Department of Genetics, School of Basic Medical Sciences, Tianjin Medical University, Tianjin, China; 2 Department of Cell Biology, School of Basic Medical Sciences, Tianjin Medical University, Tianjin, China; 3 Beijing Neurosurgical Institute, Beijing Tiantan Hospital, Capital Medical University, Beijing, China; 4 Department of Neuro-oncology, Tianjin Huanhu Hospital, Tianjin, China; 5 Department of Maxillofacial and Otorhinolaryngology Oncology, Tianjin Medical University Cancer Institute & Hospital, Tianjin, China; 6 Key Laboratory of Cancer Prevention and Therapy, Tianjin Cancer Institute, National Clinical Research Center of Cancer, Tianjin, China; 7 Department of Pathology, Tianjin Huanhu Hospital, Tianjin, China; 8 Department of Medicine, The University of Oklahoma Health Sciences Center, Oklahoma City, OK USA; 9 Department of Surgery, The University of Oklahoma Health Sciences Center, Oklahoma City, OK USA

**Keywords:** Chemotherapy, Tumour biomarkers

## Abstract

Temozolomide (TMZ) therapy offers minimal clinical benefits in patients with glioblastoma multiforme (GBM) with high EGFR activity, underscoring the need for effective combination therapy. Here, we show that tonicity-responsive enhancer binding protein (NFAT5) lysine methylation, is a determinant of TMZ response. Mechanistically, EGFR activation induces phosphorylated EZH2 (Ser21) binding and triggers NFAT5 methylation at K668. Methylation prevents NFAT5 cytoplasm interaction with E3 ligase TRAF6, thus blocks NFAT5 lysosomal degradation and cytosol localization restriction, which was mediated by TRAF6 induced K63-linked ubiquitination, resulting in NFAT5 protein stabilization, nuclear accumulation and activation. Methylated NFAT5 leads to the upregulation of MGMT, a transcriptional target of NFAT5, which is responsible for unfavorable TMZ response. Inhibition of NFAT5 K668 methylation improved TMZ efficacy in orthotopic xenografts and patient-derived xenografts (PDX) models. Notably, NFAT5 K668 methylation levels are elevated in TMZ-refractory specimens and confer poor prognosis. Our findings suggest targeting NFAT5 methylation is a promising therapeutic strategy to improve TMZ response in tumors with EGFR activation.

## Abstract (web summary)

EGFR amplification can affect temozolomide sensitivity in glioblastomas (GBM). Here the authors show that EGFR signalling mediates lysine methylation of NFAT5 which contributes to reduced efficacy of temozolomide in GBM.

## Introduction

Glioblastoma multiforme (GBM) is a highly aggressive cancer of the central nervous system, with a median survival time of only 12.2 months^1–7^. Temozolomide (TMZ), which is the mainstream chemotherapeutic agent for advanced GBM, radiotherapy with concomitant and TMZ offers median overall survival (OS) of 16 to 18 months^8,9^. EGFR amplification and its constitutively active mutant, EGFRvIII (deletion of exons 2-7)^10^, is a driving force in promoting GBM tumorigenesis and TMZ resistance^11–14^. However, the combination of anti-EGFR therapy with TMZ therapy yielded limited therapeutic benefits^15^. Therefore, it is critical to uncover the mechanisms underlying GBM pathogenesis and identify promising therapeutic targets to develop an efficacious combination therapy.

Tonicity-responsive enhancer binding protein (NFAT5, TonEBP), activated by hypertonicity, is initially identified as a central transcriptional regulator of osmoprotective response and the immune response^16,17^. Tonicity-regulated NFAT5 nuclear import depends on binding to the importin β1 (IMB1)^18^. Recent studies indicate that NFAT5 is overexpressed and associated with poor prognosis in GBM, pancreatic cancer, and melanoma^19–21^. In particular, NFAT signaling was among the top two upregulated signaling pathways in TMZ-treated U87/EGFRvIII expressing cells^22^. However, the regulatory mechanism governing NFAT5 activation independent of tonicity stimuli and its role in TMZ response remains elusive in GBM.

Lysine methylation of proteins is involved in subcellular localization, protein-protein interactions, and protein stability^23–26^. A series of studies reveal that histone methyl transferases such as EZH2 can modulate the signaling pathway activity through direct methylation of non-histone proteins^27–29^. Abnormal lysine methylation of non-histone proteins induced by growth factors such as EGF, leads to their hyperactivity and cancer progression^28,30,31^. Thus, targeting lysine methylation of non-histone protein provides a therapeutic strategy for cancer treatment. However, whether and how lysine methylation regulates NFAT5 activity remains unknown.

In this study, we show that NFAT5 undergoes methylation at K668 by EZH2 upon EGFR activation. And we elucidate the underlying mechanism of the lysine methylation-dependent regulation of NFAT5 subcellular localization and function. Furthermore, we identify a role for NFAT5 K668 methylation in modulating the response to TMZ both in orthotopic xenografts and PDX models. High levels of NFAT5 K668 methylation are associated EGFR activity, TMZ refractory and poor survival in GBM. These findings suggest that targeting NFAT5 methylation could be a promising therapeutic strategy to enhance the TMZ response in patients with GBM exhibiting upregulated EGFR activity.

## Results

### NFAT5 expression is up-regulated in TMZ-resistant GBM specimens and positively correlates with p-EGFR expression

**Fig. 1:**
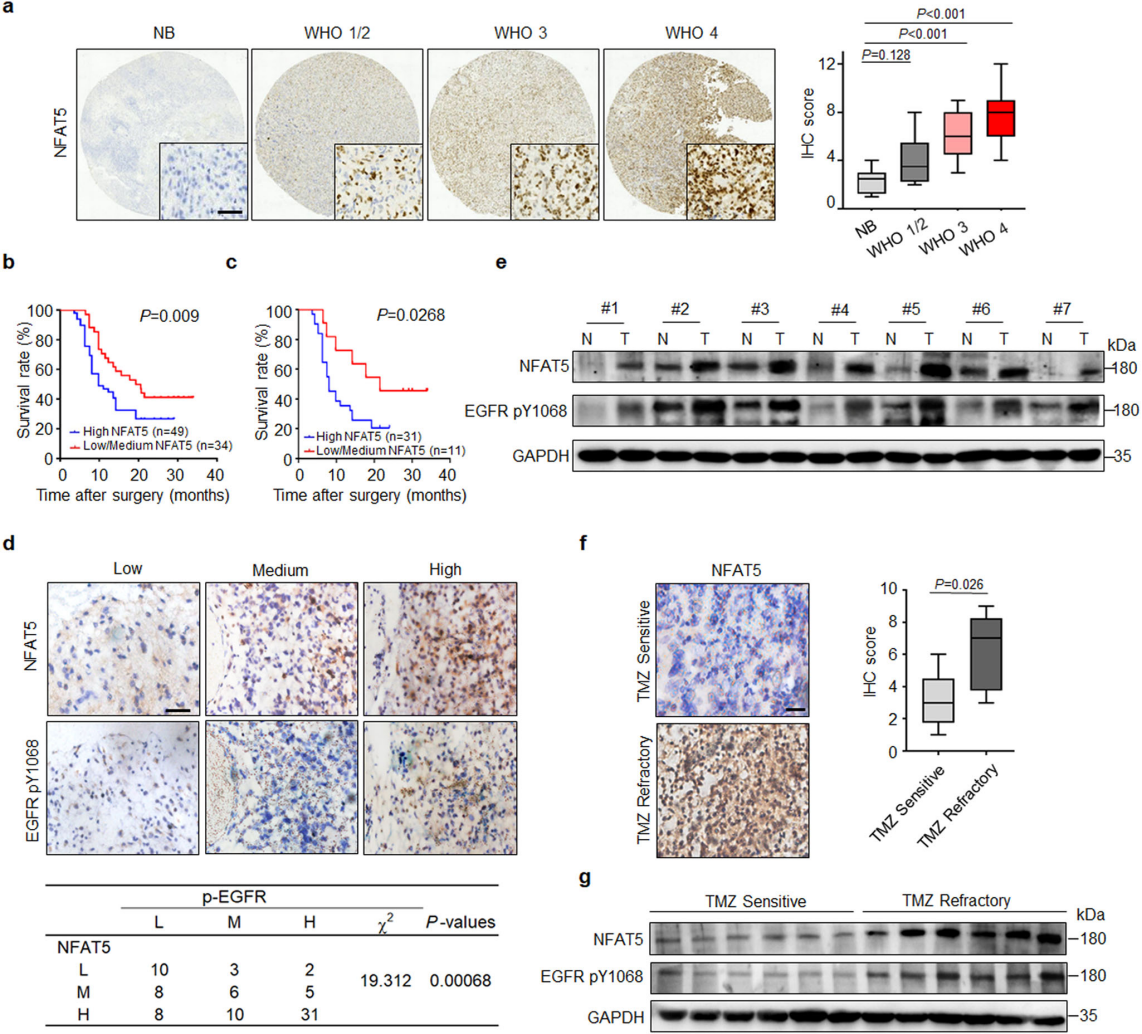
NFAT5 expression is upregulated in TMZ-resistant GBM specimens and positively correlates with p-EGFR expression. **a** Representative immunohistochemical (IHC) staining of NFAT5 in tumorous and adjacent non-tumorous brain (NB) tissues of patients with glioma (NB: 8 cases; WHO 1/2: 12 cases; WHO 3: 16 cases; WHO 4: 55 cases). Scale bar: 100μm. IHC analysis of NFAT5 expression at different tumor stages (right). NB (Min = 1, Q1 = 1.25, Med = 2.5, Q3 = 3, Max = 4); WHO 1/2 (Min = 2, Q1 = 2.25, Med = 3.5, Q3 = 5.5, Max = 8); WHO 3 (Min = 3, Q1 = 4.5, Med = 6, Q3 = 8, Max = 9); WHO 4 (Min = 4, Q1 = 6, Med = 8, Q3 = 9, Max = 12). **b** Kaplan–Meier survival analysis of patients with glioma. **c** Survival analysis of patients with IDH wild-type GBM based on NFAT5 expression. **d** Upper, NFAT5 and EGFR pY1068 expression in glioma tissues (*n* = 83 samples) determine by IHC staining. Scale bar: 100μm. Bottom, The association between NFAT5 and EGFR pY1068 levels in glioma tissue. L, low; M, medium; H, high. **e** The NFAT5 and EGFR pY1068 levels in clinical GBM samples and paired adjacent non-tumorous tissue. N, normal; T, tumor. **f** Representative IHC staining of NFAT5 in TMZ sensitive and resistant GBM tissues (*n* = 12 samples). Scale bar: 100μm. TMZ sensitive (Min = 1, Q1 = 1.75, Med = 3, Q3 = 4.5, Max = 6); TMZ resistant (Min = 3, Q1 = 3.75, Q2 = 7, Q3 = 8.25, Max = 9). TMZ, Temozolomide. **g** The protein levels of NFAT5 and EGFR pY1068 in the TMZ sensitive and refractory GBM specimen. (**e**, **g**) *n* = 3 independent experiments. Significance was calculated (**a**) by one-way ANOVA with LSD-t; (**d**) by chi-square test; (**f**) by unpaired two-sided Student’s *t* test. Data were presented as mean ± standard deviation. Marker unit for Western blots is kDa. Source data are provided as a Source Data file.

To determine the clinical significance of NFAT5, we analyzed the NFAT5 levels in 83 glioma specimens and adjacent non-tumor brain (NB) tissues obtained from the Tianjin Huanhu Hospital. NFAT5 expression levels were significantly elevated in GBM tissues compared with those in low-grade gliomas (LGGs) and adjacent NBs (Fig. 1a and Supplementary Table 1, 2). Moreover, IHC scores revealed that 69.1% of (38/55) GBM samples display elevated NFAT5 expression. The median survival durations of glioma patients harboring tumors with low and high NFAT5 expression were 19.9 and 9.8 months, respectively (Fig. 1b), and 21.5 and 8.1 months for patients with IDH wild-type GBM, respectively (Fig. 1c). These data suggest NFAT5 overexpression may contribute to GBM progression.

Aberrant EGFR activation contributes to GBM progression and TMZ resistance development^32^. To examine the relationship between EGFR activity and NFAT5 levels in the clinical setting, the expression of EGFR pY1068 and NFAT5 were analyzed in the 83 glioma specimens (Fig. 1d). In particular, 63.3% of (31/49) tumor samples with high NFAT5 expression exhibited a strong p-EGFR staining. Meanwhile, 66.7% of (10/15) samples with low NFAT5 expression had weak or no p-EGFR IHC signal. The expression of NFAT5 in GBM tissues was upregulated compared with adjacent NBs and positively correlated with that of p-EGFR (Fig. 1e). These results indicate that NFAT5 expression is physiologically and clinically relevant to EGFR activity in GBM.

Moreover, the expression and nuclear localization of NFAT5 were higher in TMZ-refractory samples than those in TMZ-sensitive specimens (Fig. 1f, g and Supplementary Table 3). Collectively, these results demonstrate that NFAT5 expression is upregulated in TMZ-refractory tissues and positively correlates with EGFR activity.

### NFAT5 is required for EGFR-driven tumor growth and the failure of TMZ therapy

**Fig. 2:**
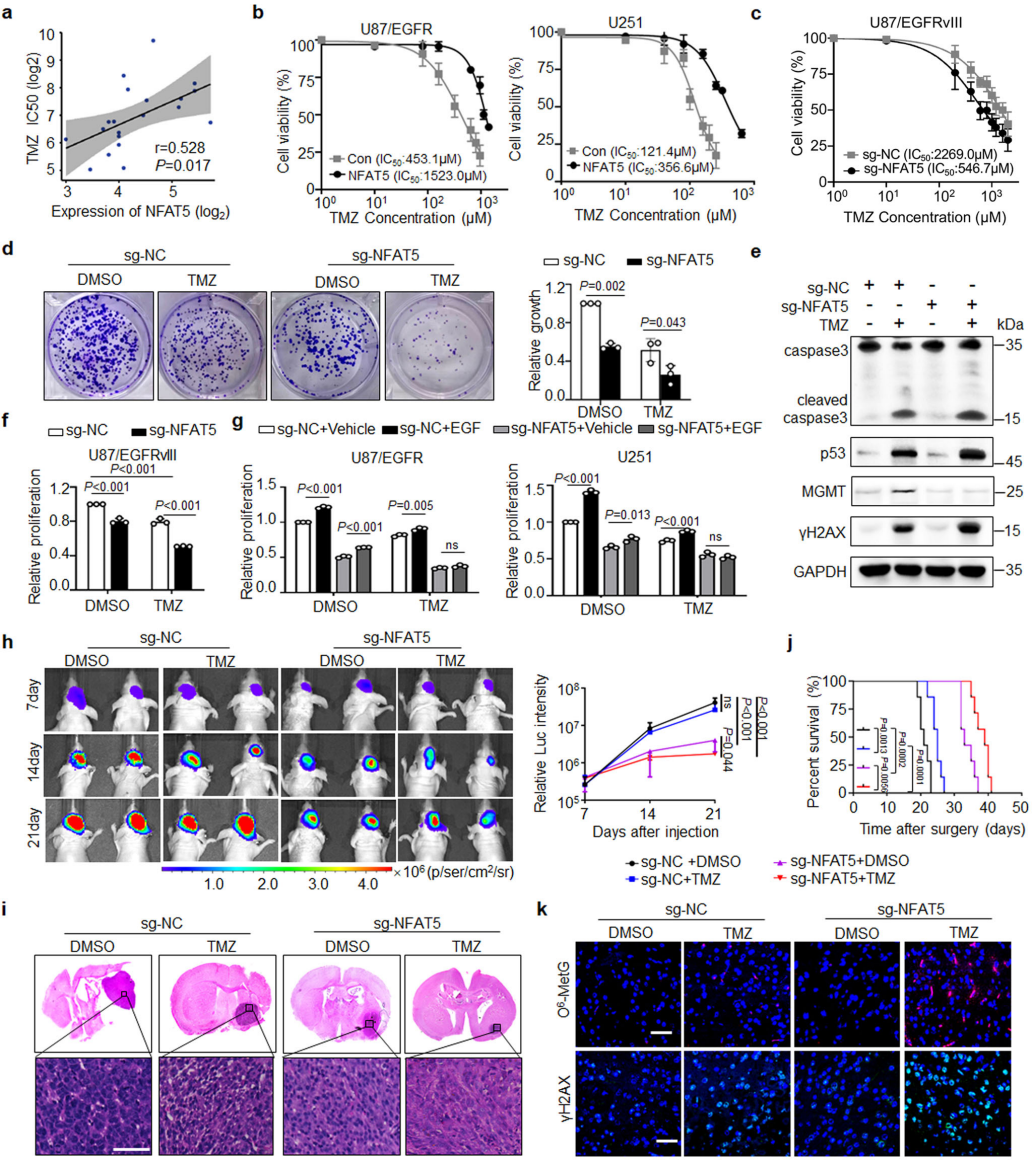
NFAT5 drives EGFR-induced tumor growth and the failure of TMZ therapy. **a** The association between NFAT5 expression and TMZ response from 20 GBM cell lines from the Genomics of Drug Sensitivity in Cancer (GDSC) database. **b**, **c** The effect of NFAT5 on TMZ efficacy at the indicated concentrations determined by CCK-8 assay. **d** Colony formation assay in U87/EGFRvIII cells expressing sg-NC or sg-NFAT5 with or without TMZ treatment (200 μM). DMSO, Dimethylsulfoxide. **e** The effect of NFAT5 on MGMT and cleaved caspase3 expression in U87/EGFR cells incubated with or without TMZ. **f** Knockout of NFAT5 reduced cell proliferation and enhanced TMZ efficacy (200 μM, 72 h) in U87/EGFRvIII cells. **g** Loss of NFAT5 attenuated EGFR activation-induced cell proliferation and TMZ refractory (200 μM, 72 h) in U87/EGFR and U251 cells. **h** The representative bioluminescence images of Balb/c nude mice with tumors derived from U87/EGFRvIII cells transfected with sg-NC or sg-NFAT5 treated with TMZ every week (*n* = 4 mice per group). **i** H&E-stained coronal brain sections of representative tumor xenograft (*n* = 7 mice/group). Scale bar: 100μm. **j** Survival of mice bearing U87/EGFRvIII tumors (*n* = 7 mice/group). **k** IF staining of O^6^-MetG and γH2AX in tumors derived from U87/EGFRvIII cells transfected with sg-NC or sg-NFAT5 treated with TMZ. *n* = 7 randomly captured field of view. Scale bar: 50 µm. (**b**–**k**) *n* = 3 independent experiments. Significance was calculated by (**a**) Pearson test; (**d**, **g**) by unpaired student’s *t* test; (**f**) by one-way ANOVA with LSD-t; (**h**) by ANOVA of repeated measurement data; (**j**) by Log-rank (Mantel–Cox) test. Data were presented as mean ± standard deviation. Marker unit for Western blots is kDa. Source data are provided as a Source Data file.

To explore the function of NFAT5 in regulating cancer drug resistance, we first obtained the TMZ response data across 505 cancer cell lines from the Genomics of Drug Sensitivity in Cancer (GDSC) database (https://www.cancerrxgene.org/). NFAT5 expression displayed significant association with the resistance to TMZ, especially in GBM cell lines (Fig. 2a and Supplementary Fig. 1a). We next examined the expression of NFAT5 in various GBM cell lines. NFAT5 expression in TMZ resistant (U87/EGFRvIII cells) was significantly upregulated compared with that in TMZ sensitive cells (U251, U87, and LN229 cells) (Supplementary Fig. 1b). To study the role of NFAT5 in regulating TMZ sensitivity, we constructed NFAT5-overexpressing and knockout stable cell lines (Supplementary Fig. 1c–f). The half-maximal inhibitory concentration (IC_50_) value of TMZ against NFAT5-overexpressing U87/EGFR and U251 cells increased by 2.93- and 2.92-fold, respectively (Fig. 2b). In contrast, loss of NFAT5 markedly enhanced the TMZ sensitivity in U87/EGFRvIII cells (Fig. 2c). Consistently, in vitro colony formation ability was reduced and the percentage of apoptotic cells were significantly increased in TMZ treated NFAT5 deficiency U87/EGFRvIII, U87/EGFR cells and U251 cells (Fig. 2d and Supplementary Fig. 1g, h). We further showed that increased apoptosis in TMZ-treated sg-NFAT5 groups was due to increased DSB accumulation shown by elevated γH2AX expression (Fig. 2e and Supplementary Fig. 1i). Moreover, NFAT5 knockdown inhibited EGFR-induced cell proliferation and TMZ resistance in U87/EGFRvIII, U87/EGFR and U251 cells (Fig. 2f, g).

Next, the role of NFAT5 in regulating the efficacy of TMZ in vivo was assessed. TMZ therapy exerted a limited tumor-inhibitory effects against sg-NC-transfected U87/EGFRvIII cells. Bioluminescent images and hematoxylin and eosin (H&E) staining demonstrated that loss of NFAT5 significantly abrogated EGFR-driven tumor growth and enhanced the sensitivity of tumors to TMZ (Fig. 2h, i). Additionally, the survival time of TMZ-treated mice harboring tumors with sg-NFAT5 transfected cells was higher than that of mice from other groups (Fig. 2j).

As an alkylating agent, the cytotoxicity of TMZ is mediated by the addition of methyl groups at O^6^ sites on guanines (O^6^-MetG) in genomic DNA^33^, result in DNA double stand breaks and eventually cell apoptosis^34^. TMZ alone induced a weak accumulation of O^6^-MetG level in mice bearing sg-NC tumors, whereas loss of NFAT5 significantly restored TMZ-induced O^6^-MetG expression (Fig. 2k). Double-strand break accumulation was found in mice bearing sg-NTAT5 tumors analyzed by γH2AX expression. These results suggest that NFAT5 is required for EGFR activation-induced tumor growth and limited TMZ efficacy by reducing the alkylating function of TMZ.

### EGF induces NFAT5 lysine methylation and activation dependent on EZH2

**Fig. 3:**
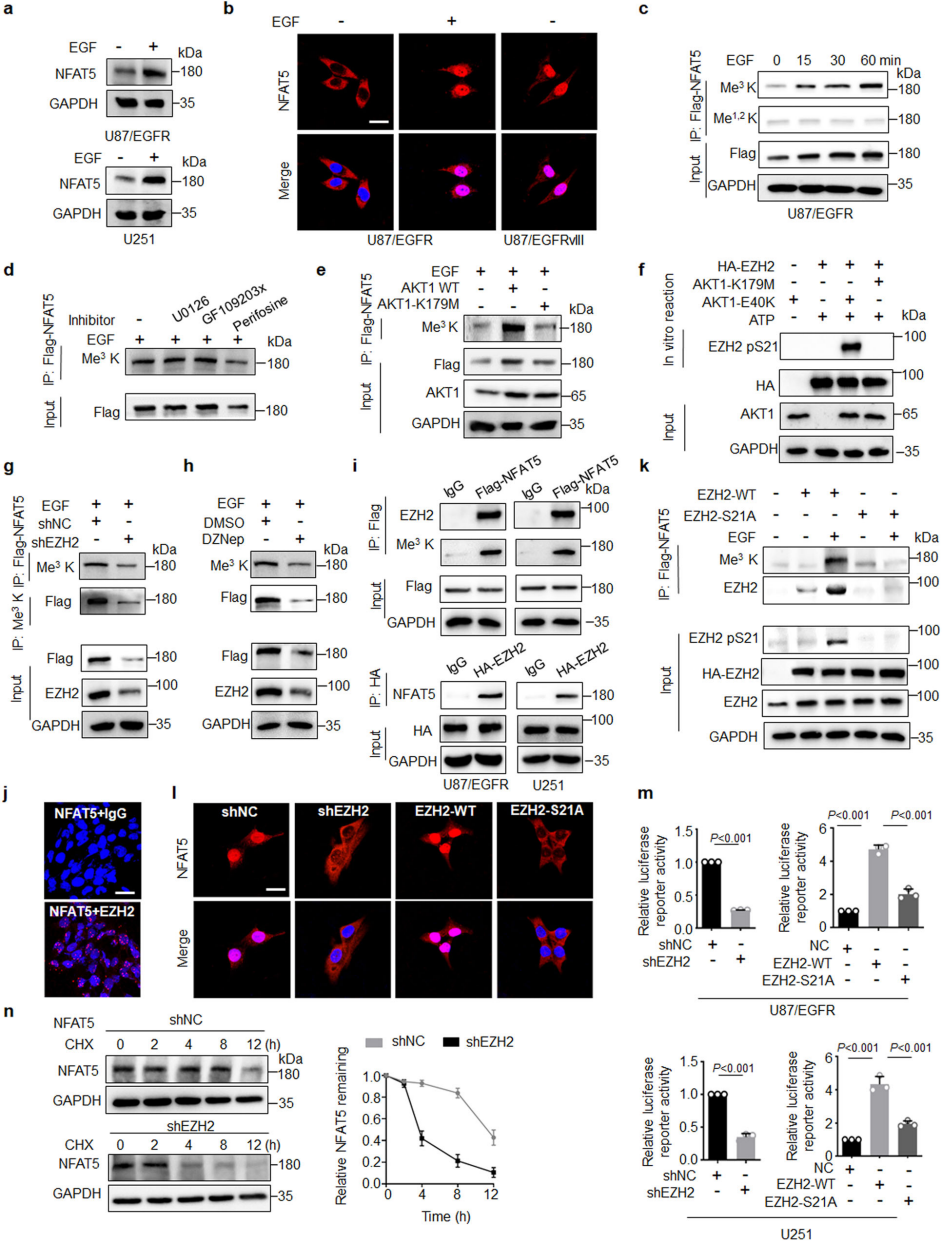
EGF induces NFAT5 lysine methylation and activation dependent on EZH2. **a** NFAT5 protein levels in U87/EGFR and U251 cells upon EGF stimulation. **b** IF staining the subcellular location of NFAT5 in U87/EGFRvIII cells and U87/EGFR cells with or without EGF treatment. Scale bar: 20 μm. One representative field of *n* = 30 independent cells was captured. **c** The effect of different incubation time of EGF treatment on the expression and lysine methylation levels of NFAT5 in U87/EGFR cells. **d** Levels of NFAT5 lysine methylation in U87/EGFR cells treated with 10 μM PKCα (GF109203X), 10 μM MEK1 (U0126), or 10 μM AKT1 (perifosine) inhibitors for 24 h followed by EGF (100 ng/mL) for 15 min. **e** AKT1 kinase activity was required for EGF induced NFAT5 tri-lysine methylation. U251 cells transduced with lentiviral vectors harboring Flag-tagged kinase-dead mutant AKT-K179M or AKT-WT sequences. **f** In vitro kinase assay revealed that AKT1-mediated EZH2 phosphorylation at Ser21 in U251 cells. **g** Knockdown of EZH2 by shRNA or a small molecule inhibitor (DZNep) treatment (**h**) reduced EGF-induced NFAT5 lysine methylation in U87/EGFR cells. DZNep, 3-Deazaneplanocin A. **i** Co-immunoprecipitation assay was performed to examine EZH2 as an interactor of NFAT5 in U87/EGFR and U251 cells. **j** The results of the in situ proximity ligation assay revealed the direct interaction between NFAT5 and EZH2 in U251 cells. Scale bar: 20μm. One representative field of *n* = 30 independent cells was captured. **k** EZH2 phosphorylation at Ser21 was critical for EZH2/NFAT5 interaction and NFAT5 methylation upon EGF stimulation. **l** IF staining of the subcellular location of NFAT5 transfected with shNC, shEZH2, EZH2-WT and EZH2-S21A in U251 cells treated with EGF. Scale bar: 20μm. One representative field of *n* = 30 independent cells was captured. **m** NFAT5 TAD reporter luciferase activity was measured in cells transfection with shNC, shEZH2 or NC, EZH2-WT and EZH2-S21A cells upon EGF treatment. **n** Degradation of NFAT5 was assessed by CHX treatment transfected with shNC or shEZH2 in U251 cell. Right, quantification of the NFAT5 intensity. **a**–**n** *n* = 3 independent experiments. Significance was calculated by (**m**) by one-way ANOVA with LSD-t. Data were presented as mean ± standard deviation. Marker unit for Western blots is kDa. Source data are provided as a Source Data file.

As EGFR activation is critical for tumorigenesis and TMZ resistance, the effect of EGF stimulation on NFAT5 expression and activation was examined. NFAT5 protein levels were upregulated in U87/EGFR and U251 cells upon EGF stimulation (Fig. 3a), whereas no significant difference in NFAT5 mRNA levels was detected (Supplementary Fig. 2a). Western blotting and immunofluorescent staining revealed enhanced nuclear abundance of NFAT5 in EGF-treated cells (Fig. 3b and Supplementary Fig. 2b). Higher nuclear NFAT5 levels were observed in the EGFR-constitutively active U87/EGFRvIII cells than in U87/EGFR cells (Fig. 3b and Supplementary Fig. 2c). Additionally, EGF stimulation increased NFAT5 protein stability in U87/EGFR and U251 cells (Supplementary Fig. 2d). These results indicate that EGF regulates NFAT5 expression through post-translational modification.

Methylation of lysine residues affect subcellular localization and protein stability^35–37^. EGF stimulation induced NFAT5 lysine tri-methylation (Me^3^ K) but not mono or demethylation (Me^1,2^ K) in both U87/EGFR and U251 cells (Fig. 3c and Supplementary Fig. 2e, f). To explore the downstream pathway involved in NFAT5 methylation in response to EGF stimuli, cells were treated with several small-molecule inhibitors of the EGFR downstream pathway, including perifosine (AKT1 inhibitor), U0126 (MEK1 inhibitor), and GF109203X (PKCα inhibitor), respectively. Treatment with perifosine markedly blocked EGF-induced NFAT5 methylation in both U87/EGFR and U251 cells (Fig. 3d and Supplementary Fig. 2g, h). Consistently, knockdown of AKT1 by shRNA mitigated NFAT5 methylation induced by EGF (Supplementary Fig. 2i). The levels of NFAT5 methylation in the AKT1 kinase-dead K179M mutant-transfected cells was markedly lower than that in the AKT1 WT-transfected cells, suggesting that AKT1 kinase activity was required for EGF-mediated NFAT5 methylation (Fig. 3e).

Previous studies reported that Ser21 of EZH2 is a substrate of AKT1^38^. Phosphorylation of EZH2 at Ser 21 exerts pro-tumorigenic functions in a non-histone methylation-dependent manner in several types of cancer^29,39^. An in vitro phosphorylation assay using constitutively active E40K mutant and kinase-dead mutant K179M confirmed that AKT1-mediated EZH2 phosphorylation at Ser21 (Fig. 3f). EZH2 knockdown by shRNA or treatment with DZNep (a small molecule inhibitor of EZH2), largely reduced EGF-induced NFAT5 lysine methylation (Fig. 3g, h and Supplementary Fig. 2j, k). The results of Co-IP and proximity ligation assays (PLA) confirmed that NFAT5 physical interacted with EZH2 in U87/EGFR and U251 cells (Fig. 3i, j). EGF-induced NFAT5 methylation and the association with EZH2 in EZH2 WT but not S21A mutation (Fig. 3k). Furthermore, NFAT5 methylation was detected in the EZH2 S21D expressing cells similar as EGF-treated EZH2 WT cells (Supplementary Fig. 2l). Additionally, the H3K27me^3^ enrichment in the promoter region of NFAT5 was not affected in EZH2 knockdown treated cells (Supplementary Fig. 2m). These results suggest that EZH2 is the critical methyltransferase for NFAT5 lysine methylation upon EGFR activation.

We next investigated the effect of EZH2 on NFAT5 subcellular localization and activation. Western blotting and immunofluorescent staining revealed that knockdown of EZH2 reduced EGF-induced NFAT5 nuclear translocation, whereas expression of EZH2 WT, but not S21A mutant rescued NFAT5 nuclear distribution (Fig. 3l and Supplementary Fig. 3a). We then used an EZH2 mutant in which the NLS motif was deleted and failed to enter the nucleus, this mutation inhibited EGF-induced nuclear translocation of NFAT5, indicating that nuclear EZH2 was the key factor for NFAT5 to enter the nucleus (Supplementary Fig. 3b, c). EZH2-WT increased NFAT5 transcriptional activity, but S21A mutant groups could not, suggesting that phosphorylation of EZH2 at S21 was required for NFAT5 activity (Fig. 3m). Moreover, knockdown of EZH2 obviously decreased NFAT5 protein stability and half-life time, suggesting that EZH2 was involved in the NFAT5 protein stability (Fig. 3n). Collectively, these findings imply that EZH2 is required for NFAT5 lysine methylation and activation in response to EGF stimulation.

### Methylation at K668 determines NFAT5 nuclear localization and activation

**Fig. 4:**
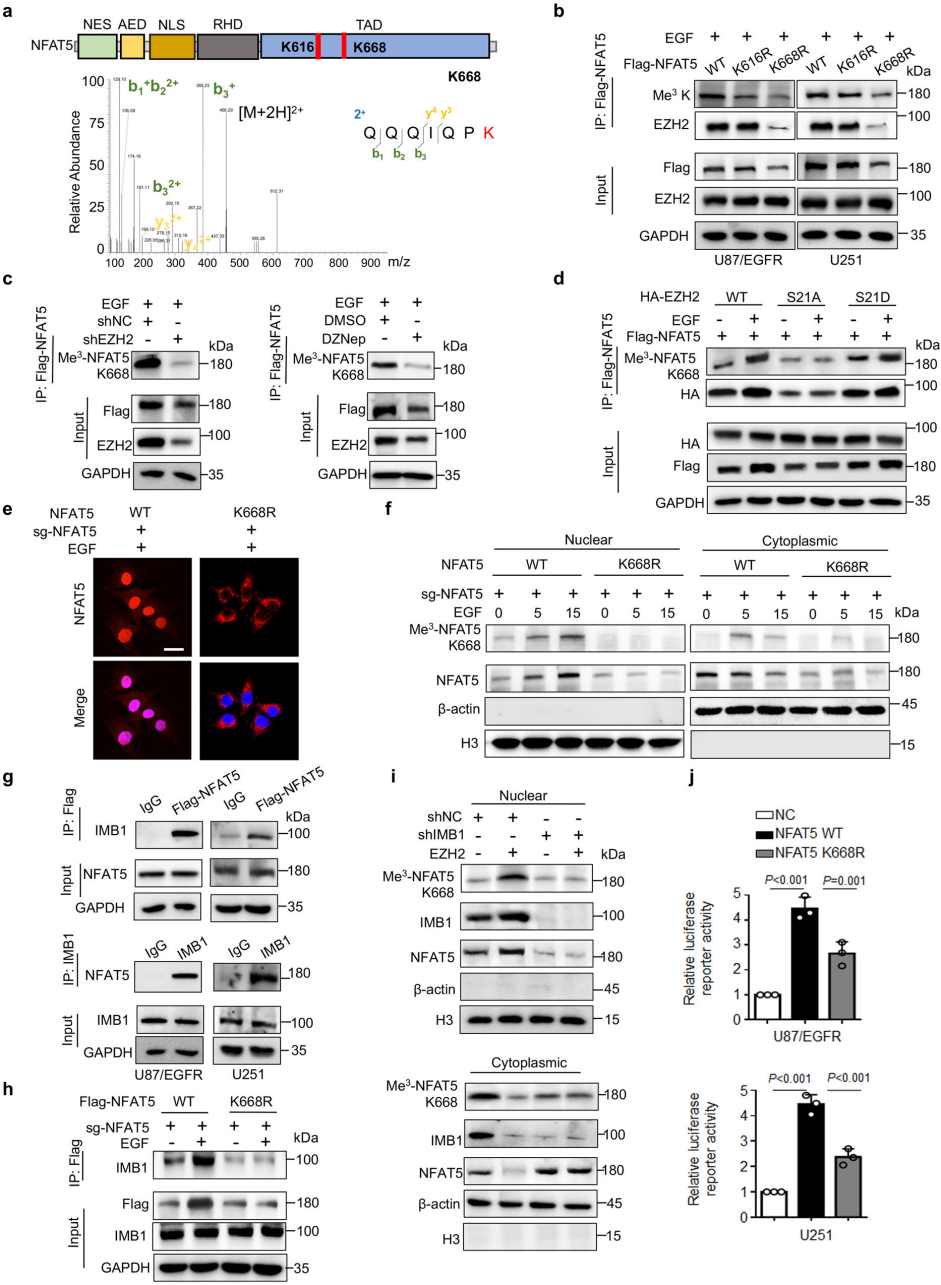
Methylation at K668 determines NFAT5 nuclear localization and activation. **a** Identification of lysine methylation site of NFAT5 by LC-MS/MS, corresponding to one of two methylation sites, K668. **b** K668 (but not K616) mitigated EGF-induced NFAT5 tri-methylation and EZH2/NFAT5 interaction. **c** Knockdown of EZH2 by shRNA or DZNep mitigated EGF-induced NFAT5 K668 methylation in U87/EGFR cells. **d** Expression of Me^3^-NFAT5 K668 and the association between NFAT5 and EZH2 was analyzed in HA-tagged EZH2 WT, S21A, S21D expressing U251 cells. **e** IF staining the subcellular location of NFAT5 in U251 cells stably expressing NFAT5 WT or K668R mutant. Scale bar: 20μm. One representative field of *n* = 30 independent cells was captured. **f** The nuclear and cytosolic fractions of U87/EGFR cells expressing NFAT5 WT or K668R mutant treated with EGF at different time were subjected to immunoblotting analysis. **g** Co-IP assay was performed to examine IMB1 as an interactor of NFAT5 in the U87/EGFR and U251 cells. **h** The association of NFAT5 and IMB1 was examined in NFAT5 WT or K668R U87/EGFR cells in the presence of EGF or not. **i** The nuclear and cytosolic expression of NFAT5 and Me^3^-NFAT5 K668 was detected in EZH2 overexpressing cells transfected with shNC or shIMB1. **j** NFAT5 TAD reporter luciferase activity was measured in cells transfected with NFAT5 WT or K668R. (**b**–**j**) *n* = 3 independent experiments. Significance was calculated by (**j**) one-way ANOVA with LSD-t. Data were presented as mean ± standard deviation. Marker unit for Western blots is kDa. Source data are provided as a Source Data file.

We used mass spectrometry analysis to identify the specific methylation sites of NFAT5. Two lysine residues (K616 and K668), which were located in the C-terminal transactivation domain (TAD), were identified in NFAT5 (Fig. 4a and Supplementary Fig. 4a). Moreover, both residues were highly conserved from Gallus Gallus to humans (Supplementary Fig. 4b). The methylation status of NFAT5 K668R mutant, but not the K616R mutant, was significantly lower than those of NFAT5 WT upon EGF stimulation (Fig. 4b and Supplementary Fig. 4c). In contrast to NFAT5 WT, K668R mutants was disassociated with EZH2.

Next, a K668-specific methylation antibody against NFAT5 was generated to identify K668 tri-methylation of NFAT5 (anti-Me^3^-NFAT5 K668), which was validated by dot blot, ELISA and IF staining (Supplementary Fig. 4d–f). Analysis with pan-lysine tri-methylation and anti-Me^3^-NFAT5 K668 revealed that EGF-induced NFAT5 K668 tri-methylation in NFAT5 WT cells, which was disappeared in NFAT5 K668R overexpressing cells (Supplementary Fig. 4g). Additionally, NFAT5 K668 methylation expression was impaired in cells infected with shEZH2 or small molecule inhibitors DZNep or EPZ-6438 treatment^40^ (Fig. 4c and Supplementary Fig. 4h–k). Of note, the NFAT5 K668 tri-methylation in EZH2 WT but not S21A was markedly enhanced in response to EGF stimulation. Expression of constitutively active EZH2 S21D mutant was sufficient to induce NFAT5 K668 methylation to a degree similar to that observed in response to EGF treatment in U87/EGFR and U251 cells (Fig. 4d and Supplementary Fig. 4l). Moreover, knockdown of EZH2 resulted in a decrease of 57.6% in the tri-methylation occupancy of the Lys668 residue relative to con cells, based on the lysine methylation stoichiometric analysis of LS-MS/MS data (Supplementary Fig. 4m). Meanwhile, elevated NFAT5 K668 methylation levels were observed in the EGFR-constitutively active U87/EGFRvIII cells, compared to EGF treated U87/EGFR cells (Supplementary Fig. 4n).

We then sought to examine whether K668 methylation influence NFAT5 subcellular localization. Biochemical fractionations/western blot analysis and immunofluorescence revealed that EGF treatment induced the nuclear accumulation of NFAT5 WT but not K668R mutant expressing cells (Fig. 4e and Supplementary Fig. 5a, b). NFAT5 K668 methylation was detected in the cytosol peaked at 5 min in response to EGF treatment, and then recruited to the nucleus (Fig. 4f and Supplementary Fig. 5c). These results indicate that EZH2-mediated NFAT5 methylation at K668 initially occurred in the cytosol, and is crucial for NFAT5 nuclear accumulation upon EGF stimulation.

Recent studies have shown that IMB1, a nuclear transport receptor, is required for NFAT5 nucleus translocation^18^. Co-IP confirmed that NFAT5 interacted with IMB1 in U251 and U87/EGFR cells (Fig. 4g). As expected, EGF stimulation increased the binding of NFAT5 and IMB1, while the interaction was greatly weakened in NFAT5 K668R mutant cells (Fig. 4h and Supplementary Fig. 5d). Consistently, knockdown of IMB1 reduced EZH2-induced the NFAT5 nuclear accumulation (Fig. 4i and Supplementary Fig. 5e). Meanwhile, EZH2 S21A reduced the binding of NFAT5 and IMB1, compared to EZH2 WT cells (Supplementary Fig. 5f). Notably, NFAT5 K668R mutation showed decreased transcriptional activity (Fig. 4j). These results suggest that methylation at K668 promotes NFAT5 binding to IMB1, result in NFAT5 nuclear localization and activation upon EGFR activation.

### Methylation protects NFAT5 from ubiquitin-mediated lysosome degradation by attenuating its binding to E3 ligase TRAF6

**Fig. 5:**
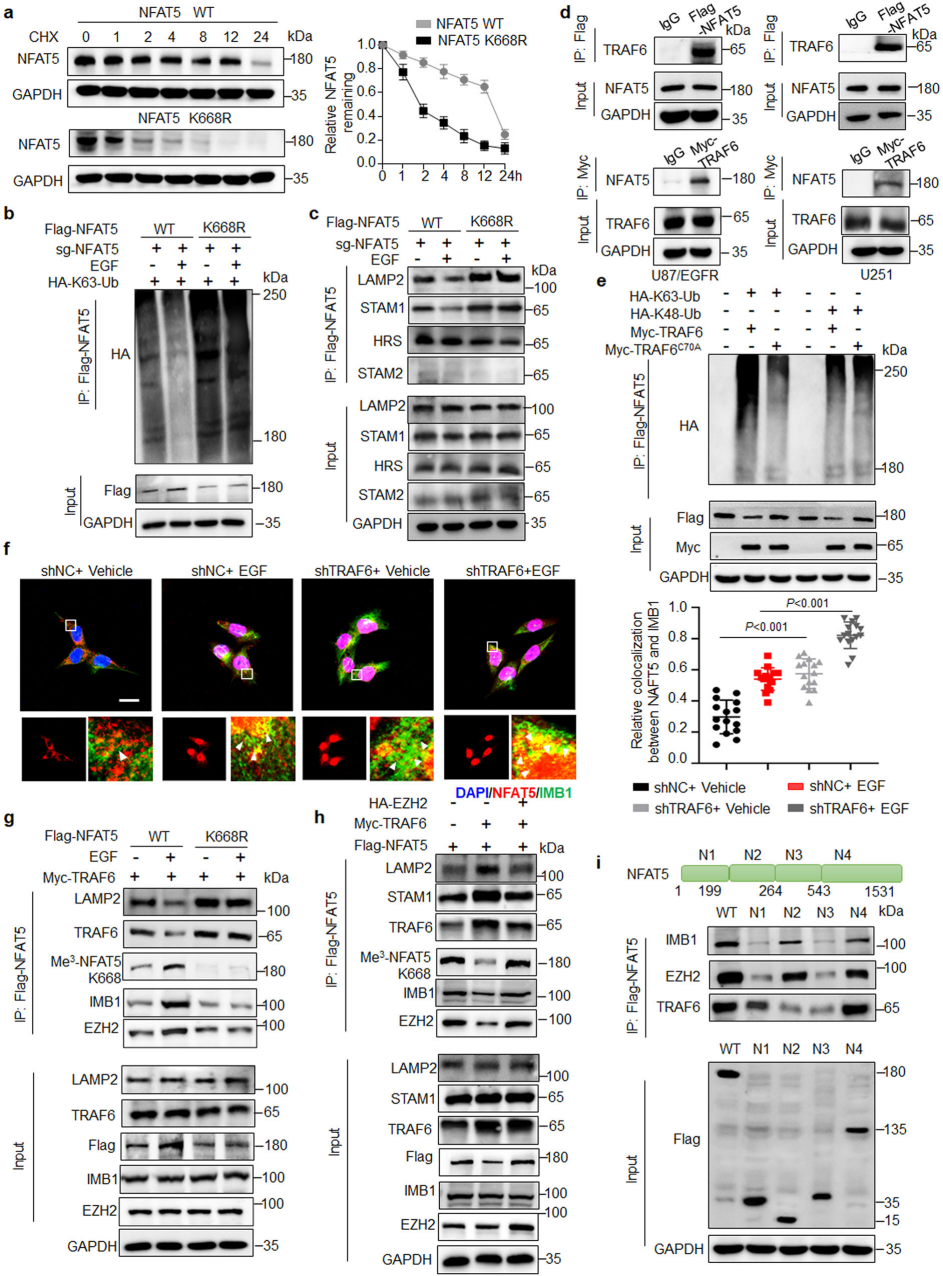
Methylation protects NFAT5 from ubiquitin-mediated lysosome degradation and cytosol restriction by attenuating its binding to E3 ligase TRAF6. **a** Degradation of NFAT5 was assessed by CHX treatment in NFAT5 WT or K668R mutant expressing U87/EGFR cells. Right, quantification of the NFAT5 intensity. CHX, cycloheximide. **b** NFAT5 K63-linked ubiquitination and the association between NFAT5 and ESCRT-0 complex (**c**) were examined in U251 expressing NFAT5 WT and K668R mutant cells. **d** Co-IP analysis of the interaction between TRAF6 and NFAT5 in U87/EGFR and U251 cells. **e** TRAF6 activation was required for NFAT5 K63 but not K48-linked ubiquitination. **f** IF staining of NFAT5 subcellular distribution and co-localization between NFAT5 and IMB1 in shNC or shTRAF6 transfected U251 cells in the presence of EGF or not. Scale bar: 20μm. Quantitative results from 15 cells per group are reported. **g** The interaction between NFAT5 and TRAF6, EZH2, LAMP2 as well as IMB1 was examined in TRAF6 overexpressing cells in the absence or presence of EGF stimulation. **h** The association of NFAT5 with TRAF6, EZH2, LAMP2, STAM1 as well as IMB1 was examined in TRAF6 overexpressing cells in the absence or presence EZH2 transfection. **i** Identification of the essential domains required for interactions of NFAT5 with TRAF6, EZH2 and IMB1. Diagrammatic representation of NFAT5 and its truncated forms. **a**–**i** *n* = 3 independent experiments. Marker unit for Western blots is kDa. Source data are provided as a Source Data file.

As expected, K668R mutant reduced NFAT5 protein stability and half-life time (Fig. 5a and Supplementary Fig. 6a). Meanwhile, the degradation of NFAT5 K668R was inhibited by concanamycin-A (ConA, an inhibitor of lysosome), but not MG132 (an inhibitor of proteasome) or 3-Methyladenine (3-MA, an inhibitor of autophagy) treatment (Supplementary Fig. 6b). U87/EGFR and U251 cells were pretreated with concanamycin-A, the methylation levels of NFAT5 at K668 was significantly reduced in EZH2 knockdown cells by shRNA or EPZ6438 treatment, while the total levels of NFAT5 was not changed, suggesting that EZH2 methylated NFAT5 methylation at site K668 (Supplementary Fig. 6c, d). NFAT5 K668R mutant increased the association between NFAT5 with LAMP2 (Fig. 5c and Supplementary Fig. 6e). Consistently, the NFAT5/LAMP2 complex formation was enhanced by shEZH2 transfected or DZNep treated cells (Supplementary Fig. 6f).

Lysine methylation modulates protein stability through regulating ubiquitination modification^41,42^. K668R mutant obviously increased the ubiquitination of NFAT5 (Supplementary Fig. 6g). Similar results were detected in cells with EZH2 knockdown by shRNA or DZNep treatment (Supplementary Fig. 6h, i). Furthermore, EGF reduced K63- but not K48-linked ubiquitination of NFAT5, whereas such ubiquitination was markedly induced in K668R mutant expressing cells (Fig. 5b and Supplementary Fig. 6j, k). We further demonstrated that the K63-linked ubiquitination of NFAT5 was observed only in the cytosol (Supplementary Fig. 6l). These results show that K668 methylation of NFAT5 increases protein stability by inhibiting K63-linked ubiquitin-mediated lysosomal degradation in the cytoplasm.

The ubiquitin-dependent lysosomal degradation requires the endosomal sorting complexes required for transport (ESCRT) complexes, including HRS, STAM1 and STAM2^43^. Co-IP results indicated that the association of NFAT5 with STAM1, but not HRS nor STAM2, was decreased in NFAT5 K668R cells compared with NFAT5 WT cells (Fig. 5c). Furthermore, knockdown of STAM1 rescued NFAT5 degradation induced by methylation blockade (Supplemental Fig. 6m), further confirming the requirement of the ESCRT-0 complex in lysine methylation-induced NFAT5 stabilization.

To identify the putative E3 ligase that catalyzed NFAT5 K63-linked ubiquitination, liquid chromatography-tandem mass spectrometry/mass spectrometry (LC-MS/MS) analysis was performed and TRAF6 was revealed as a potential E3 ligase of NFAT5 (Supplementary Fig. 7a). The results of co-IP confirmed that NFAT5 physical interacted with TRAF6 in U251 and U87/EGFR cells (Fig. 5d). TRAF6 significantly promotes the K63 but not K48-linked ubiquitination of NFAT5. In contrast, mutation of TRAF6 in the RING domain (TRAF6^C70A^), which was required for E3 ligase activity, lost the ability to ubiquitinate NFAT5 (Fig. 5e). Moreover, TRAF6 mediated cytoplasmic K63-linked ubiquitination of NFAT5 in U251 cells (Supplementary Fig. 7b). These results suggest that TRAF6 is a K63 specific ubiquitin ligase for NFAT5. Moreover, the association between NFAT5 and TRAF6 was markedly increased in NFAT5 K668R cells upon EGF stimulation, compared to the NFAT5 WT cells. IF staining showed that the localization signal of NFAT5 and TRAF6 was seen by yellow staining in most of NFAT5 K668R mutant cells, but not NFAT5-WT cells (Supplementary Fig. 7c). Meanwhile, knockdown of TRAF6 could restore NFAT5 expression in NFAT5 K668R mutant or shEZH2 cells (Supplementary Fig. 7d, e). Therefore, these results indicate that K668 methylation of NFAT5 is a critical signal to mask the recognition of E3 ligase TRAF6 to NFAT5, consequently protecting NFAT5 from ubiquitin-mediated lysosome degradation.

### NFAT5 K668 methylation interferes TRAF6 induced NFAT5 cytosol localization restriction

TRAF6-mediated ubiquitination is also involved in protein subcellular distribution^44,45^. Biochemical fractionations/western blot results demonstrated that knockdown of TRAF6 enhanced the nuclear localization of NFAT5 in the absence of EGF stimulation (Supplementary Fig. 7f). To elucidate the mechanism by which TRAF6 controls NFAT5 nuclear distribution, we determined whether TRAF6 mediated NFAT5 K63-linked ubiquitination orchestrated the interaction between NFAT5 with IMB1. Immunofluorescence staining demonstrated that knockdown of TRAF6 significantly increased the co-localization signal of NFAT5 and IMB1 (Fig. 5f). Overexpressing TRAF6 blocked the association of NFAT5 and IMB1 in the absence of EGF treatment. And the binding affinity of NFAT5 to IMB1 was restored upon EGF stimulation in NFAT5 WT cells, but not in K668R mutant expressing cells (Fig. 5g). NFAT5 interacted with TRAF6 at serum-starved condition, and then declined rapidly with the prolongation of EGF treatment. On the contrary, EGF stimulation induced the binding of NFAT5 to EZH2 and IMB1 (Supplementary Fig. 7g). NFAT5 K668R mutant displayed strong association of TRAF6 and marked reduction in interacting with EZH2 and IMB1 under basal conditions and or EGF treatment. Consistently, TRAF6 induced the association of NFAT5 with LAMP2 and STAM1 in control cells, which was significantly reduced in EZH2 overexpressing cells (Fig. 5h). These results suggested that TRAF6 induced NFAT5 cytosol localization restriction through disrupting the interaction between NFAT5 and IMB1.

To underlying the mechanism involved in NFAT5 K668 methylation reduced the binding affinity of TRAF6, we constructed fragmented plasmids of different domains of NFAT5. Results of Co-IP showed that both N2 and N4 domain were the critical domain for NFAT5 and EZH2 as well as IMB1 binding. TRAF6 bounds to NFAT5 at the N1 and N4 domain (Fig. 5i). N1 domain and N2 domain are reported to modulate NFAT5 nuclear export and import, respectively. N4 domain, where K668 site was located, was critical for NFAT5 activation^46^. These results indicate that EZH2-induced NFAT5 methylation disrupts the interaction between NFAT5 and TRAF6, leading to NFAT5 binding to IMB1 and translocating to the nucleus. These results suggest that TRAF6 triggers NFAT5 ubiquitination reduces the interaction between NFAT5 and IMB1, thus keeping NFAT5 away from the nucleus. NFAT5 K668 methylation by EZH2 disrupts the association with TRAF6 and NFAT5, result in NFAT5 nuclear translocation.

### K668 methylation is required for NFAT5-induced limited TMZ efficacy by enhanced *MGMT* transcription

**Fig. 6:**
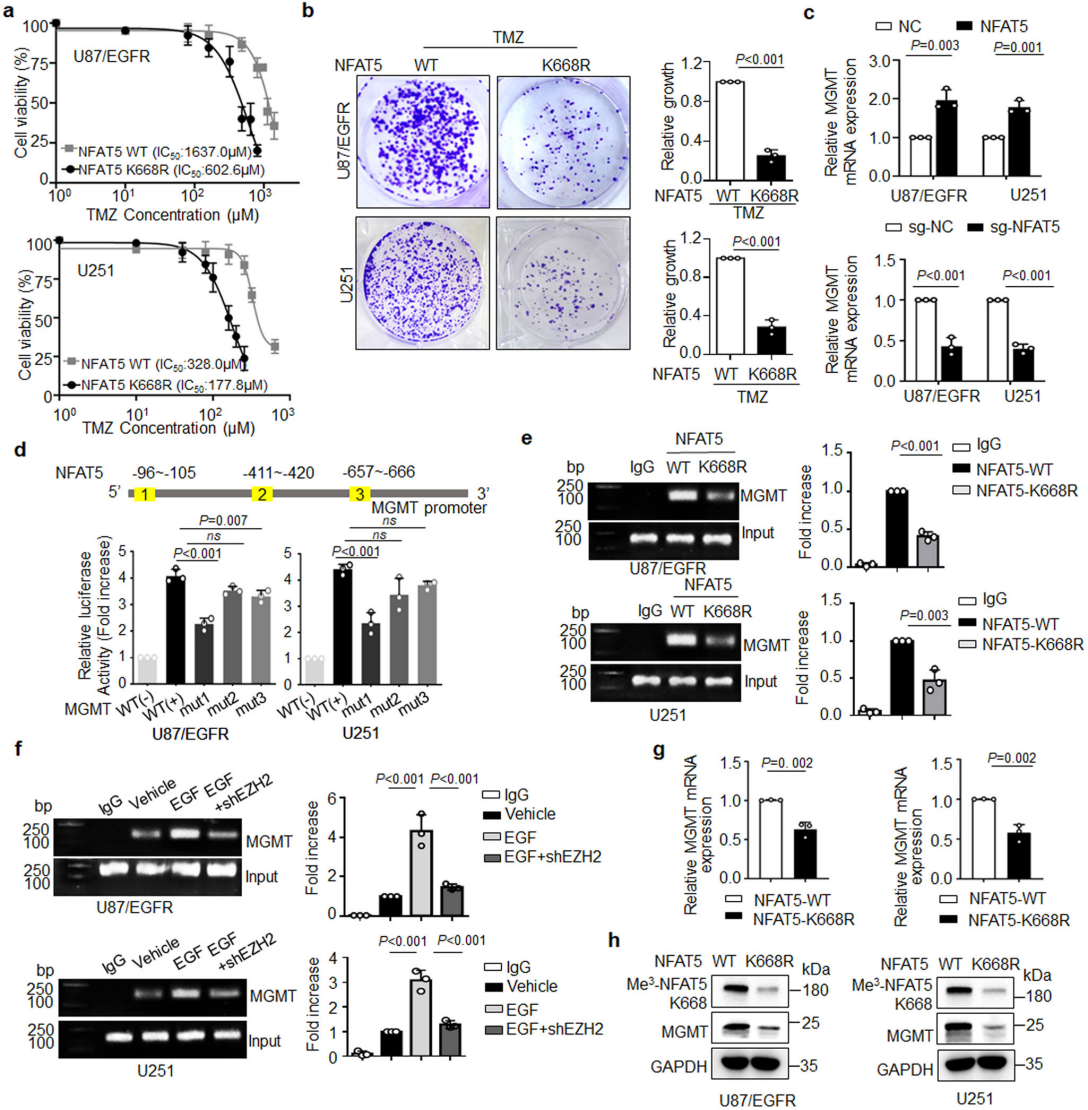
K668 methylation is required for NFAT5 induced limited TMZ efficacy by enhanced *MGMT* transcription. **a** CCK-8 assay analysis revealed the effect of NFAT5 K668 methylation on TMZ efficacy at the indicated concentrations for 72 h. **b** Colony formation assay in cells expressing NFAT5 WT or K668R with TMZ treatment. **c** The effect of NFAT5 on MGMT mRNA levels in U87/EGFR and U251 cells. **d** The results of the dual-luciferase reporter assay revealed that MGMT was a direct transcriptional target of NFAT5. **e** ChIP assay demonstrated that NFAT5 K668R mutation reduced NFAT5 binding affinity to *MGMT* promoter, compare to the NFAT5 WT cells. **f** CHIP assay revealed that *EZH2* knockdown by shRNA decreased the binding affinity of NFAT5 to the promoter of *MGMT*. **g** The mRNA and protein levels (**h**) of MGMT in U87/EGFR and U251 cells expressing NFAT5 WT or K668R mutation. **a**–**h** *n* = 3 independent experiments. Significance was calculated by (**b**, **c**, **g**) unpaired Student’s *t* test; (**d**, **e**, **f**) by one-way ANOVA with LSD-t. Data were presented as mean ± standard deviation. Marker unit for Western blots is kDa. Marker size for CHIP is bp. Source data are provided as a Source Data file.

To unveil the significance of NFAT5 K668 methylation in modulating the efficacy of TMZ therapy, the growth-inhibitory efficacy of TMZ against NFAT5 WT and K668R mutant-transfected cells was examined. The IC_50_ of TMZ in NFAT5-K668R cells was 1.83- and 2.85-fold lower than those against NFAT5 WT transfected U251 and U87/EGFR cells, respectively (Fig. 6a). Additionally, in vitro colony formation ability in the TMZ treated NFAT5 K668R expressing cells were largely reduced, compared to NFAT5 WT in U87/EGFR, U251 and U87/EGFRvIII cells (Fig. 6b and Supplementary Fig. 8a). Increased γH2AX expression was detected in TMZ treated NFAT5 K668R cells (Supplementary Fig. 8b).

O^6^-MeG can be removed by methylguanine methyltransferase (MGMT; direct repair) or tolerated in mismatch repair-deficient (MMR-) tumors^47–50^. We analyzed the effect of NFAT5 on MGMT and MMR-related genes in both U251 and U87/EGFR cells. The results revealed that overexpression of NFAT5 enhanced the mRNA level of MGMT, whereas knockdown of NFAT5 reduce the mRNA level of MGMT (Fig. 6c). However, no significant change was observed in MMR-related genes (Supplementary Fig. 8c). Several potential binding sites of NFAT5 in the promoter region of MGMT were identified based on bioinformatic analysis of HumanTFDB database. Luciferase reporter assay activity indicated that MGMT was a direct transcriptional target of NFAT5 (Fig. 6d).

To identify the other downstream gene of NFAT5 in modulating TMZ efficacy, mRNA sequencing was performed to analyze the signaling pathways associated with NFAT5 upregulation. Differentially expressed genes (DEGs) were enriched in “Apoptosis” and “ECM-receptor interaction” pathways based on the KEGG pathway analysis (Supplementary Fig. 9a). The integrin signaling pathway was reported to be critical for tumor progression, metastasis, and TMZ resistance therapy in GBM^51,52^. Furthermore, NFAT5 regulates integrin-mediated cell adhesion and invasion^53^. Hence, the regulatory effect of NFAT5 on the key members of the integrin signaling pathway in GBM cells was examined. NFAT5 overexpression increased mRNA and protein levels of *ITGB1*, whereas NFAT5 deficiency reduced *ITGB1* expression. However, NFAT5 did not affect the levels of *ITGB3* or *ITGB6* (Supplementary Fig. 9b, c).

ITGB1 was reported to induce TMZ resistance in a p53-dependent manner^51^. Knockdown of ITGB1 in U87/EGFR and U251 cells reduced IC_50_ of TMZ by 3.50- and 1.83-fold, respectively (Supplementary Fig. 9d). Next, the regulatory effects of NFAT5 on TMZ resistance through *ITGB1* were examined. The IC_50_ of TMZ against ITGB1 knockdown U87/EGFR and U251 cells overexpressing NFAT5 decreased by 3.52- and 2.64-fold, respectively (Supplementary Fig. 9e, f). Overexpression of NFAT5 inhibited TMZ-induced p53 expression, whereas knockdown of ITGB1 rescued the p53 activity resulting from NFAT5 overexpression (Supplementary Fig. 9g). Luciferase reporter assay indicated that ITGB1 was a direct transcriptional target of NFAT5 (Supplementary Fig. 9h).

ChIP-PCR data showed that NFAT5 K668R mutation significantly reduced the binding of NFAT5 to the promoter of *MGMT* and *ITGB1* (Fig. 6e and Supplementary Fig. 10a). Consistently, knockdown of EZH2 impaired EGF-induced the increased the binding of NFAT5 to the promoter of *MGMT* and *ITGB1* (Fig. 6f and Supplementary Fig. 10b, c). Additionally, the H3K27me^3^ enrichment in the promoter region of ITGB1 was not affected in EZH2 knockdown-treated cells (Supplementary Fig. 10d). Moreover, the mRNA and protein levels of MGMT and ITGB1 were significantly reduced in NFAT5 K668R mutant transfected cells, compared to the NFAT5 WT (Fig. 6g, h and Supplementary Fig. 10e, f). Collectively, these results suggest that NFAT5 methylation at K668 by EZH2 reduces the alkylating function of TMZ by enhancing the transcription of *MGMT* and *ITGB1*.

### Inhibition of NFAT5 K668 methylation improves TMZ efficacy in orthotopic xenografts and PDX models

**Fig. 7:**
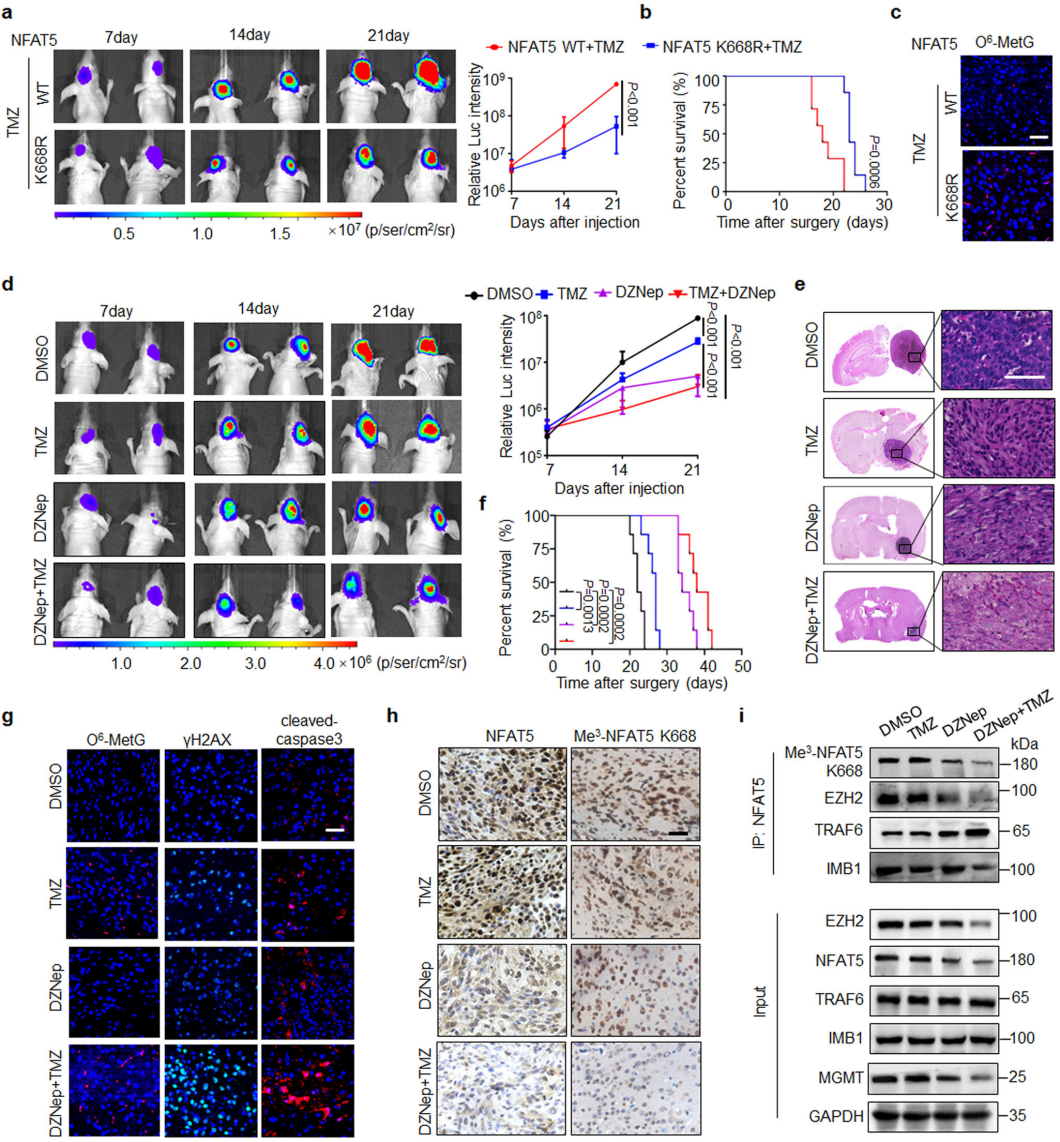
Inhibition of NFAT5 K668 methylation improves TMZ efficacy in vivo. **a** The representative bioluminescent images of Balb/c nude mice harboring tumors derived from NFAT5 WT or K668R mutant-transfected U87/EGFRvIII cells treated with TMZ therapy every week (*n* = 4 mice per group). **b** Kaplan–Meier survival curves of mice shown in (**a**), *n* = 7 mice/group. **c** Representative images of IF staining of O^6^-MetG in tumors derived from NFAT5 WT or K668R mutant-transfected U87/EGFRvIII cells treated with TMZ therapy. *n* = 5 randomly captured field of view. Scale bar: 50 µm. **d** The representative bioluminescence images of mice bearing tumors from U87/EGFRvIII cells treated with TMZ, DZNep, alone or in combination (*n* = 4 mice per group). **e** Representative H&E-stained coronal brain sections of mice from experiment shown in (**d**). Scale bar: 100μm. **f** Survival of mice injected with TMZ, DZNep alone or in combination, *n* = 7 mice/group. **g** IF staining of O^6^-MetG, γH2AX and cleaved-caspase3 in tumors derived from U87/EGFRvIII cells treated with TMZ, DZNep, alone or in combination. *n* = 5 randomly captured field of view. Scale bar: 50 µm. **h** IHC staining of NFAT5 and Me^3^-NFAT5 K668 expression in the tumors from experiment shown in (**d**). Scale bar: 100μm. **i** Combination of TMZ and DZNep impaired the interaction between NFAT5 and EZH2 as well as IMB1. **a**–**i** *n* = 2 independent experiments; Significance was calculated by (**a**, **d**) ANOVA of repeated measurement data; (**b**, **f)** by Log-rank (Mantel–Cox) test; Data were presented as mean ± standard deviation. Marker unit for Western blots is kDa. Source data are provided as a Source Data file.

To examine the role of NFAT5 K668 methylation in conferring TMZ efficacy in vivo, NFAT5 WT or K668R mutant was stably overexpressed in U87/EGFRvIII cells. Bioluminescence analyses revealed that the growth of NFAT5 K668R-transfected cell-derived tumors was slower than that of NFAT5 WT cell-derived tumors (Fig. 7a). Meanwhile, the NFAT5 K668R mutant prolonged survival time, compared with NFAT5 WT group mice (Fig. 7b). Reduced Ki67 and elevated cleaved caspase 3 as well as γH2AX expression were detected in TMZ treated NFAT5 K668R cell-derived tumors (Supplementary Fig. 11a, b). Moreover, stronger fluorescence signal of O^6^-MetG was observed in mice bearing NFAT5 K668R transfected U87/EGFRvIII tumors, compared to NFAT5 WT tumor cells (Fig. 7c). This indicates that NFAT5 K668R cells exhibited increased sensitivity to TMZ.

To validate these findings in vivo, an orthotopic to GBM tumor model derived by U87/EGFRvIII cells was employed to test the anti-tumor effect of DZNep/TMZ combined regimen. The combination therapy reduced the tumor size and prolonged survival time, compared to the single TMZ-treated mice (Fig. 7d–f). Meanwhile, increased cleaved caspase3 and reduced Ki67 signals were detected in DZNep/TMZ combination treatment group (Fig. 7g and Supplementary Fig. 11c). Consistently, TMZ combined with DZNep treatment increased the expression levels of O^6^-MetG and γH2AX compared to the single TMZ treatment group (Fig. 7g). Moreover, combination therapy largely reduced Me^3^-NFAT5 K668 levels and increased the association between NFAT5 and TRAF6, compared with single TMZ treatment (Fig. 7h, i).

**Fig. 8:**
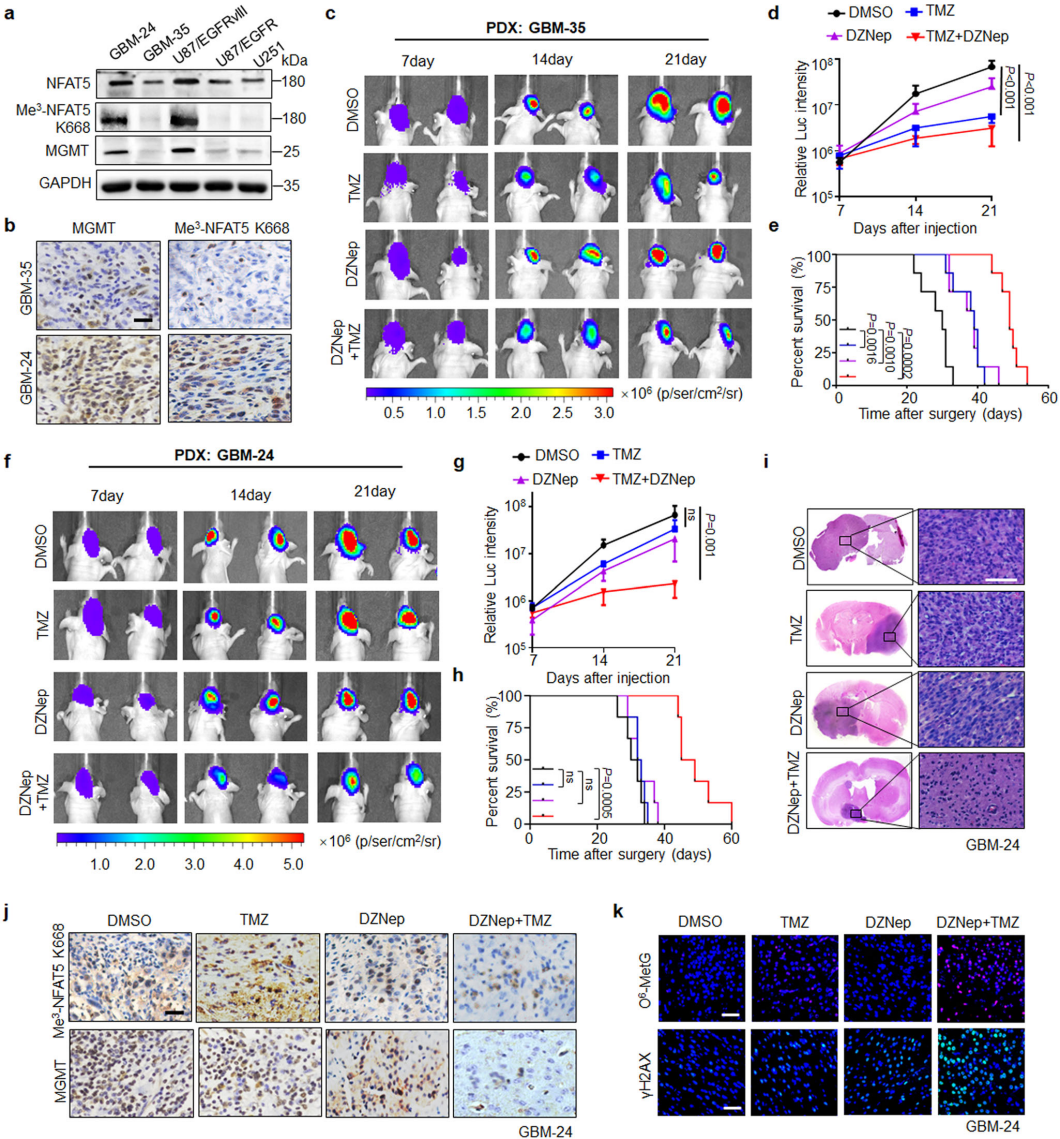
NFAT5 K668 methylation status predicts TMZ efficacy in PDX models. **a** The protein expression of NFAT5, Me^3^-NFAT5-K668 and MGMT in two PDX GBM cells lines, GBM-24 and GBM-35. **b** IHC staining of Me^3^-NFAT5-K668 and MGMT in tumors from mice orthotopically xenografted with GBM-35 and GBM-24 cells. Scale bar: 100μm. **c** Representative bioluminescent images of nude mice harboring GBM-35 tumors (*n* = 4 mice per group). **d** Tumor burden examined by bioluminescence imaging. **e** Kaplan–Meier survival curves of mice shown in (**c**), *n* = 7 mice/group. **f** Effects of TMZ, DZNep, alone or in combination on tumor growth in mice harboring GBM-24 derived tumors. **g** Tumor burden examined by bioluminescence imaging (*n* = 4 mice per group). **h** Kaplan–Meier survival curves of mice shown in (f), *n* = 6 mice/group. **i**, H&E-stained coronal brain sections of mice from experiment shown in (**f**). Scale bar: 100μm. **j** IHC staining of Me^3^-NFAT5 K668 and MGMT expression in the GBM-24 tumors from each group. Scale bar: 100μm. **k** IF staining of O^6^-MetG and γH2AX in tumors derived from GBM-24 cells treated with TMZ, DZNep, alone or in combination. Scale bar: 50 µm. **a** *n* = 3 independent experiments; (**a**–**c**, **e, f**, **h**–**k**) *n* = 2 independent experiments; Significance was calculated by (**d**, **g**) ANOVA of repeated measurement data; (**e**, **h)** by Log-rank (Mantel–Cox) test; Data were presented as mean ± standard deviation. Marker unit for Western blots is kDa. Source data are provided as a Source Data file.

We further evaluated the anti-tumor effect of combination therapy in the PDX models with different Me^3^-NFAT5 K668 levels (Fig. 8a, b). TMZ treatment reduced the tumor size and induced O^6^-MetG accumulation in lower Me^3^-NFAT5 K668 expression GBM-35 tumors (Fig. 8c-e and Supplementary Fig. 12a–c). The efficacy of TMZ was limited in GBM-24 tumors with higher Me^3^-NFAT5 K668 expression levels, whereas TMZ combined with DZNep inhibited tumor growth and prolonged the survival time of mice (Fig. 8f–i). Consistently, reduced Ki67 staining and increased cleaved caspase3 expression were detected in the combination therapy (Supplementary Fig. 12d, e). More importantly, protein levels of Me^3^-NFAT5 K668 and MGMT were remarkably decreased in GBM-24 tumors upon combination treatment, compared to TMZ treatment alone (Fig. 8j and Supplementary Fig. 12f). TMZ alone failed to induce O^6^-MetG accumulation, the addition of DZNep significantly increased TMZ induced O^6^-MetG levels (Fig. 8k). These results indicate that Me^3^-NFAT5 K668 is a master regulator of tumor progression and TMZ response, targeting NFAT5 K668 methylation as an effective therapeutic strategy to improve TMZ response in GBM.

### NFAT5 K668 methylation is positively correlated with p-EGFR Y1068 and p-EZH2 S21 expression, TMZ refractory and poor prognosis in patients with GBM

**Fig. 9:**
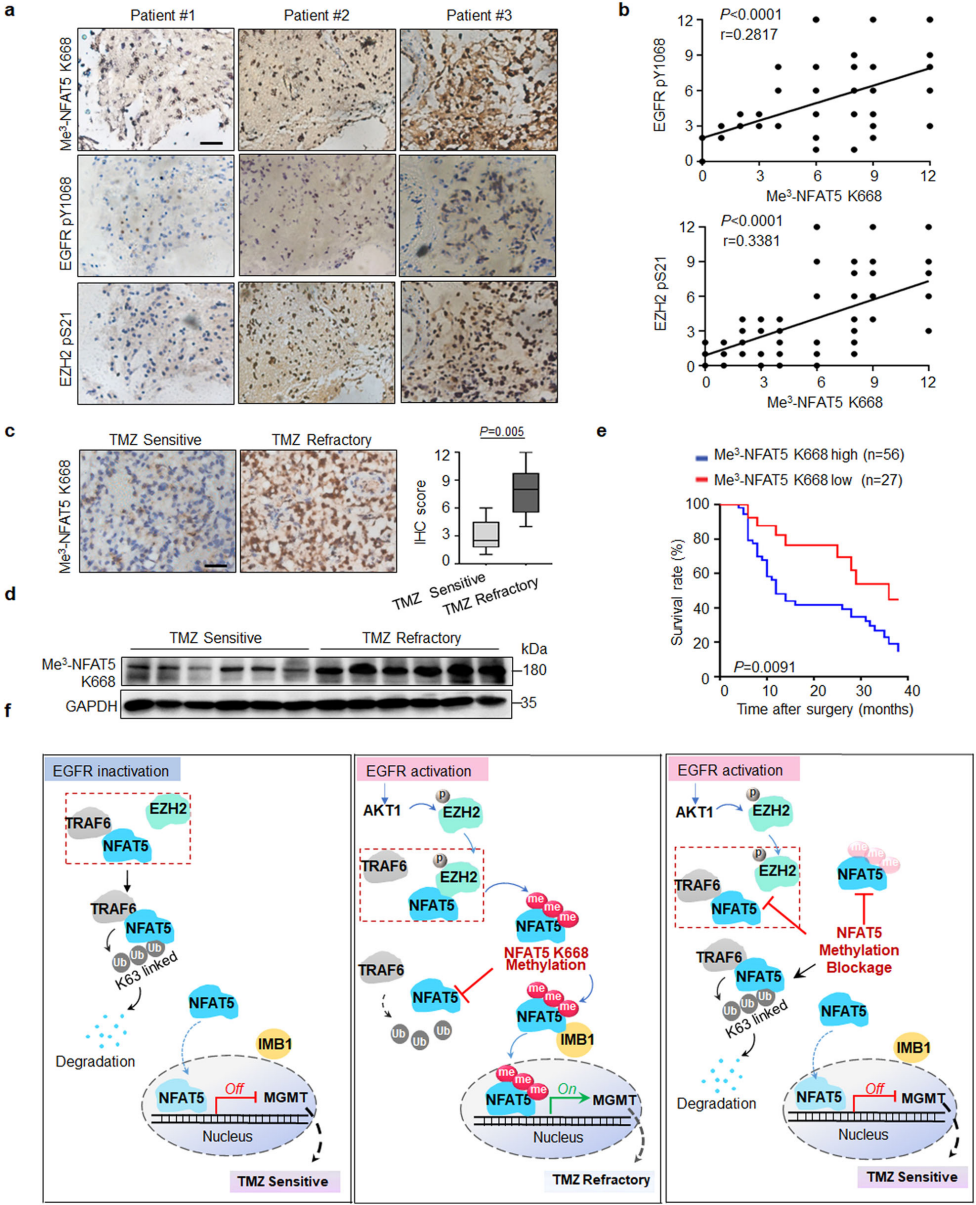
NFAT5 K668 methylation is positively correlated with EGFR pY1068 and EZH2 pS21 expression, TMZ refractory and poor prognosis in patients with GBM. **a** IHC staining of Me^3^-NFAT5 K668, EZH2 pS21 and EGFR pY1068 expression in glioma specimens (*n* = 83 samples). Scale bar: 100μm. **b** IHC stains were scored and the correlation was carried out by Pearson correlation test. **c** Representative IHC-staining of Me^3^-NFAT5 K668 in TMZ-sensitive and resistant GBM tissues (*n* = 12 samples). Scale bar: 100μm. TMZ sensitive (Min = 1, Q1 = 1.75; Med = 2.5; Q3 = 4.5 Max = 6); TMZ resistant (Min = 4, Q1 = 5.5, Med = 8, Q3 = 9.75, Max = 12). **d** Protein expression of Me^3^-NFAT5 K668 in TMZ sensitive and resistant clinical GBM samples. **e** Kaplan–Meier survival analysis of patients. Overall survival of patients with high Me^3^-NFAT5 K668 expression was shorter. **f** A proposed model illustrating lysine methylation of NFAT5 drives tumor progression and TMZ refractoriness in GBM. NFAT5 undergoes K668 methylation by EZH2 upon EGF stimulation, resulting in NFAT5 nuclear translocation and activation. Methylation disrupts NFAT5 binding to E3 ligase TRAF6, which is crucial for NFAT5 K63-linked ubiquitination mediated lysosomal degradation and cytosol localization restriction, leading to NFAT5 stabilization, nuclear accumulation and activation. Nuclear NFAT5 increased the induction of MGMT, a transcriptional target of NFAT5, which is required for unfavorable TMZ response. This suggests the development of strategies involving NFAT5 K668 methylation blockage for enhancing TMZ response in GBM. (**d**) *n* = 3 independent experiments; Significance was calculated by (**b**) Pearson test, two-sided; (**c**) by unpaired two-sided Student’s *t* test. Data were presented as mean ± standard deviation. Marker unit for Western blots is kDa. Source data are provided as a Source Data file.

To determine the clinical significance of EGFR/EZH2 dependent NFAT5 lysine methylation in glioma, we performed IHC analysis to examine the expression and correlation between EZH2 pS21 and Me^3^-NFAT5 K668 as well as EGFR pY1068 levels in 83 human glioma specimens. A total of 57.1% (32/56) glioma specimens with high NFAT5 K668 methylation levels displayed enhanced protein expression of EZH2 pS21. Meanwhile, 62.5% (35/56) glioma specimen with high NFAT5 K668 methylation levels displayed enhanced protein expression of EGFR pY1068 (Fig. 9a). These findings suggest that the expression of Me^3^-NFAT5 K668 is positively correlated with EGFR pY1068 and EZH2 pS21 in glioma (Fig. 9b).

Next, we found elevated Me^3^-NFAT5 K668 expression and nuclear abundance in TMZ-resistant, compared with sensitive GBM specimens (Fig. 9c, d). To further determine the clinical relevance of Me^3^-NFAT5 K668 level to the prognosis of GBM patients, we analyzed Me^3^-NFAT5 K668 levels and survival duration. The median survival of the GBM patients with low Me^3^-NFAT5 K668 levels is 36 months, while the median survival is 12 months in high-Me^3^-NFAT5 K668 GBM patients (Fig. 9e). This indicates that NFAT5 K668 methylation level is a robust and independent predictor of survival for patient with GBM.

## Discussion

Unfavorable TMZ response remains the leading challenge for GBM patients with aberrant EGFR activation^11,54,55^. This study uncovered that lysine methylated NFAT5, an osmoprotective responsive transcription factor, is a determinant of EGFR-driven tumor progression and the response to TMZ in GBM, reinforcing the key role of lysine methylation of non-histone proteins and demonstrating its potential as a therapeutic target. We found that EGFR activation orchestrated sequential PTM events involving lysine methylation and ubiquitination to control NFAT5 nuclear localization and activation.

The main mechanisms of TMZ drug resistance are MGMT overexpression or DNA repair deficiency^34,56^. In this study, we demonstrated that MGMT was a direct transcriptional target of NFAT5. EGFR activation facilitates NFAT5 interaction with EZH2 and triggers NFAT5 methylation at K668. Methylation results in NFAT5 stabilization, nuclear accumulation and activation, leading to transcriptional upregulation of *MGMT* (Fig. 9f). These results clearly support the notion that the previously uncharacterized EGFR/EZH2/NFAT5 axis identified may explain the high probability of tumorigenesis and why only a small fraction of GBM patients respond to TMZ. The abundant K668 methylation of NFAT5 was elevated in TMZ-resistant GBM specimens. Moreover, increased levels of NFAT5 K668 methylation was correlated with EGFR activity and conferred poor prognosis in patient with GBM. Thus, this study provides clinical and mechanistic evidence demonstrating that NFAT5 methylation is critical for EGFR-driven tumorigenesis and poor TMZ efficacy in GBM.

NFAT5 regulates the osmotic pressure balance through regulating multiple downstream genes^17^. However, the mechanism underlying the regulation of NFAT5 nuclear translocation and protein stability during cancer progression, especially upon growth factor stimulation, remains unclear. We identify TRAF6 E3 ligase as a critical cytosolic gatekeeper to keep NFAT5 away from nucleus and routed to lysosomal degradation under the serum-starved condition. Mechanistically, we show that TRAF6 interacts with cytosolic NFAT5 and triggers K63-linked ubiquitination of NFAT5, which prevents NFAT5 from binding to IMB1, consequently restrict NFAT5 cytosol localization. Moreover, TRAF6 induced NFAT5 polyubiquitination, thereby inducing its association with the core components of the ESCRT-0 complex STAM1 and facilitated NFAT5 lysosome-mediated degradation. EZH2 binds to the NFAT5 in a competitive manner with TRAF6 upon EGF stimulation. Therefore, NFAT5 K668 methylation serves as a molecular switch that regulates NFAT5 nucleus translocation and stability in GBM in response to EGFR activation.

EZH2 is mainly reported to function as a histone methyltransferase through its SET domain^57,58^. EZH2 as a methyltransferase modulates Erα and PP2A that are associated with targeted therapy resistance, which modulating tamoxifen resistance in breast cancer^59,60^. Our findings identify NFAT5 as a non-histone substrate of EZH2. We found a positive correlation between the protein expression of p-EZH2 S21 and NFAT5 K668 methylation in GBM specimens. We expanded the understanding of EZH2 substrates and its biological function. Therefore, the elucidation of the effect of non-histone lysine methylation on chemo-resistance might contribute to clinical application to improve the survival of patient with GBM.

In summary, we showed that NFAT5 K668 methylation is a key event regulating NFAT5 hyperactivation and the response to TMZ therapy. These findings provide a rationale for targeting NFAT5 K668 methylation combined with TMZ therapy for GBM patients with abnormal EGFR activation.

## Methods

### Statement about ethics

All research conduct in this study complies with all relevant ethical and safety regulations. The glioma tissues used in this study were obtained from Tianjin Huanhu Hospital, Tianjin, with informed written consent from patients. This study was approved by the Tianjin Huanhu Hospital Ethical Committee (EK 2019179). There was no bias in the selection of patients. 83 glioma specimens in total, including 55 GBM samples were included in this study. Gender-based analysis revealed that no differences existed in data obtained from glioma patients of different genders (Supplementary Table 1 and 2). All animal experiments were approved by the Ethics Committee of the Tianjin Medical University (TMUaMEC2017016) were performed following the guidelines for the use of laboratory animals. Not exceeding the maximum allowable tumor size/load (diameter less than 1.5 cm).

### Patients and samples

A total of 83 glioma tissue specimens were collected from the Tianjin Huanhu Hospital. The clinical stage of the tumor was determined and matched to the gender and age according to the American Joint Committee on Cancer (8th edition) guidelines. All of the samples are primary tumors. Patients received TMZ medication (75 mg/m^2^ once a day via intravenous injection) after surgery were recruited. The response to TMZ therapy was evaluated according to the Response Evaluation Criteria for Solid Tumors 1.1. Briefly, TMZ-resistant patients were those with disease progressive or stable disease within 6 months after the chemotherapy; while the TMZ-sensitive patients indicated those with recurrence > 6 months or no recurrence. The clinical formation of patients is shown in Table S3.

### Cell lines and reagents

Human GBM cell lines U87 (HTB-14) and LN229 (CRL-2611) cells were purchased from the American Type Culture Collection (ATCC). U251 (09063001) cells were purchased from Millipore. Cell lines are authenticated by PCR-based short tandem repeat (STR) profiling generated by Beijing Microread Genetics company. The U87/EGFRvIII cell line was a kind gift from Dr. Lei Han (Tianjin Medical University General Hospital, Tianjin)^61^. LN229, U251, and U87/EGFRVIII cells were cultured with Dulbecco’s modified Eagle medium, and U87 cells were cultured with Eagle’s minimum essential medium. The culture medium was supplemented with 100 U/mL penicillin, 100 μg/mL streptomycin, and 10% fetal bovine serum. The cells were cultured at 5% CO_2_ and 37 °C. Only cells cultured to the log phase and passaged ≤ 15 times were used.

GBM-35 and GBM-24, these two cell lines were derived from two patients with GBM, which were obtained from the Beijing Institute of Neurosurgery, Beijing Tiantan Hospital. Both of patients provided written informed consent for the use of specimens. GBM-35 and GBM-24 are cultured as neurospheres in DMEM/F12 medium (modified with L-glutamine, HEPES, Phenol Red) supplemented with B27 supplement (50×), bFGF/ EGF (20 ng/mL), penicillin-streptomycin (100 units/mL), non-essential amino acid (NEAA) in a humidified atmosphere of 5% CO_2_ at 37°C. TMZ (T-36587) were purchased from aladdin. DZNep (S7120), GF109203X (S7208), and U0126 (S1102) were purchased from Selleck Chemicals. EGF was acquired from Sino Biological (10605-HNAE).

### Generation of stable cells using lentiviral transfection

Stable cell lines were constructed as previously described^62^. To establish the human *NFAT5* knockout cells, the cells were transfected with lentiviral vectors harboring clustered regularly interspaced short palindromic repeats/caspase 9 sequences targeting human *NFAT5*. The target sequences were as follows: sgNFAT5 #1, 5′-CAGCTTACCACGGACAACAA-3′; sgNFAT5 #2, 5′-GGATATTGTCCACACAA CAT-3′; and sgNFAT5 #3, 5′-GCGTAGGGATATTGAAATTG-3’. The cells were seeded at 50% confluency 24-36 h before lentiviral transfection. After transfection for 16 h, the medium was removed and cells were cultured in medium containing 4 μg/mL puromycin (Solarbio) after 16 h of transfection for one week.

To generate the cells expressing NFAT5 wild-type (WT) or K668R mutant, the lentiviral vectors harboring Flag-tagged WT or K668R mutant NFAT5 sequences, which were purchased from Genechem, were transfected into sgNFAT5-transfected cells. The cells were seeded at 50% confluency 24–36 h before infection. The medium was replaced with a medium containing lentiviral vector. After transduction for 16 h, the medium was replaced with fresh medium and the transfected cells were selected with 600 µg/mL G418 (Solarbio) for 2 weeks.

To establish stable *EZH2* knockdown lines, the cells were transfected with a lentiviral short hairpin RNA (shRNA) system (Genechem) containing the following shRNA against *EZH2* (shEZH2): 5′-CCGGCCCAACATAGATGGACCAAATCTCGAGATTTGGTCCATCTATGTTGGGTTTTTG-3’. The target sequences used for shITGB1 were as follows: shITGB1 #1, 5′-GGCUCCAAAGAUAUAAAGATT-3′; shITGB1 #2, 5′-GCCUUCAAUAAA GGAGAAATT-3′; and shITGB1 #3, 5′-GGAGUUUGCUAAAUUUGAATT-3′. The target sequences used for shTRAF6 were as follows: 5′- GCAGUGCAAUGGAAUUUAUTT −3’ (shTRAF6#1), 5′-GCAAAUGUCAU CUGUGAAUTT-3′ (shTRAF6#2), 5′-CCCAGUCACACAUGAGAAUTT-3′ (shTRAF6#3) was used. After transfection for 16 h, the medium was replaced with fresh medium and the transfected cells were selected with 4 µg/mL puromycin for 7 days.

### Antibody generation

The anti-Me^3^K antibody generated by PTMBio (PTM-601) was widely used for the detection of tri-lysine methylation of proteins, but cannot be used for the identification of the specific lysine methylation site. An anti-NFAT5 K668 tri-methylation rabbit polyclonal antibody (anti-Me^3^ NFAT5 K668) was generated against the region near the K668 methylation site of NFAT5 by ABclone company based on the methylated peptide [N-QQQIQP-K(Me^3^)-AYNPET].

### Luciferase reporter assay

To evaluate transactivation activity of NFAT5-TAD, GAL4 reporter gene (PFR-LUC) and GAL4 DNA-binding domain (DBD) containing NFAT5 recombinant TAD were co-transfected into cells. After transfection (24 h), cells were stimulated with EGF and luciferase activity was measured 16 h later using the Luciferase Analysis System Kit (Promega) and the Synergy 2 Multiple Detection Microplate reader (Biotek).

### Western blotting and Co-immunoprecipitation (IP) assay

Western blotting was performed as previously described^63^. The proteins in the lysate were subjected to sodium dodecyl sulfate (SDS)-polyacrylamide gel electrophoresis, transferred to polyvinylidene fluoride membrane and blocked in 5% BSA in Tris-buffered saline for 1 h. Membranes were probed with the primary antibodies overnight at 4 °C, followed by incubation with secondary antibodies.

To perform IP, cell lysates were incubated with primary antibody overnight at 4 °C with gentle shaking, followed by incubation with Protein A/G Sepharose beads (ThermoFisher Scientific) for 2 h. Washed the immunoprecipitated protein with lysis buffer and subjected to immunoblotting analysis.

### Immunohistochemical (IHC) staining

Paraffin-embedded tissue sections were baked for 3 h at 60 °C and subjected to microwave treatment in citrate buffer for antigen retrieval. Next, the samples were incubated with 3% H_2_O_2_ for 15 min and blocked with 3% bovine serum albumin (BSA) for 1 h. The sections were incubated with primary antibodies overnight at 4 °C, followed incubated with the secondary antibodies (ZSGB-Bio, sp9001) for 30 min. Immunoreactive signals were developed using a 3,3′-diaminobenzidine kit (ZSGB-Bio). The sections were counterstained with hematoxylin.

The immunoreactive signal intensity was scored as follows: 0, negative staining; 1, weakly positive staining (light brown); 2, moderately positive staining (brown); and 3, strongly positive staining (dark brown). Additionally, the immunoreactive signals were quantified as follows: 0, negative; 1, positive cells ≤ 25%; 2, 26-50% positive cells; 3, 51-75% positive cells; and 4, positive cells > 75%. The EI value, which ranged from 0 to 12, was determined by multiplying the extent (E) and intensity (I) scores.

### Detection of apoptosis

Apoptosis was determined using flow cytometry with the Annexin V-FITC/PI Apoptosis detection kit (Elabscience, E-CK-A211). Wash the cells with ice-cold PBS, and resuspend them in Annexin binding buffer, finally reaching a concentration of 10^5^ cells/mL. The cell suspension (100 μL) was incubated with Annexin V-FITC Reagent (2.5 μL) and PI Reagent (2.5 μL) for 15 min in the dark. Add the Annexin binding buffer (400 μL) to the reaction mixture followed by analysis with the flow cytometer (BD FACSVerse).

### Promoter activity assay

The human *MGMT* promoter sequence was downloaded from the USCS genomic browser. The WT and three mutant promoter sequences of *MGMT* were cloned into the pGL3-basic plasmid and synthesized by Genechem Company (Shanghai). The NFAT5-binding sequence was predicted using the HumanTFDB database with the relative threshold set at 80%. The mutant *MGMT* promoter sequence-harboring vector was constructed using mutated binding sites of NFAT5 at the *MGMT* promoter region. The WT or mutant *MGMT* promoter-harboring vector was co-transfected with the control Renilla luciferase reporter into the cells. Luciferase activity was measured by the dual luciferase reporter gene assay system (Promega)^62^.

### Silver staining and mass spectrometry analysis

The cells were lysed in NETN lysis buffer (50 mM Tris, 0.2 mM EDTA, 1% Triton X-100, 150 mM NaCl, and complete TM protease inhibitor cocktail). Next, the lysates were incubated with anti-FLAG M2 affinity gel (Sigma) with gentle rocking at 4 °C for 3 h, followed by incubation with flag-peptides at 4 °C for 3 h. To denature the proteins, the samples were incubated at 70 °C for 10 min. The bands were visualized using silver staining with a silver staining kit (ThermoFisher Scientific). Distinct protein bands were analyzed by LC-MS/MS.

For identification of interacting proteins, a protein band visualized via silver staining was excised from an SDS-PAGE gel and digested in gel in 50 mM ammonium bicarbonate buffer containing RapiGest (Waters Corporation) overnight at 37 °C with 200 ng of modified sequencing-grade trypsin (Promega). The digested protein samples were analyzed using high-sensitivity LC-MS/MS with an Orbitrap Elite mass spectrometer (ThermoFisher Scientific).

### Mass spectrometry data analysis

Raw files were processed with the MASCOT search engine (Matrix Science, London, UK; version 2.2) against the UniProt protein database. The search parameters were set as follows: (i) Trypsin with a maximum of two missed cleavages was allowed; (ii) Precursor mass tolerance of 20 ppm and the fragment mass tolerance of 0.1 Da; (iii) Carbamidomethyl (C) was set as static modification; Oxidation (M), Methyl, Dimethyl, Trimethyl (K), and Deamidation (NQ) were set as variable modifications. The peptide and protein identifications were filtered by score ≥ 20.

### Chromatin immunoprecipitation assay (ChIP)

ChIP analysis was performed using the EZ ChIP Kit (Millipore 17-10086) based on the manufacturer’s instruction. The cells were fixed with 1% formaldehyde and neutralized with 0.125 M glycine. Next, the cells were washed with cold PBS three times and lysed in SDS lysis buffer (containing 1% SDS, 50 mM Tris HCl (pH 8.0), and 10 mM EDTA). 200-500-bp chromatin fragments were collected after sonication and pre-cleared in dilution buffer. The fragments were incubated with homologous IgG or NFAT5 antibody on a rotating platform overnight at 4 °C. The immunocomplexes were collected using protein G beads and the DNA-binding antibody fragments were eluted for real-time PCR analysis with the following primers: *ITGB1*, 5′-GTCTCACCACCCTTCGTGAC-3′ (forward) and 5′-CCTGAGTCCCGAGGCAAATC-3′ (reverse). *MGMT*, 5′-AGTGGCCAGGTGTATAGCATT-3′ (forward) and 5′- TAAGACTTCGGGAAACTCGTCT-3′ (reverse).

### RNA sequencing

Total RNA was isolated, and RNA quantification and quality assessment were performed. RNA integrity and genomic DNA contamination were tested by denaturing agarose gel electrophoresis. RNA from each sample was removed by ribosomal RNA prior to RNA-seq library construction. The cDNA library was then sequenced and 100 base pairs were subjected to a paired-end run^64^. RNA-seq reads were compared with the human reference genome using the STAR method and read removal replicates were performed using the PICARD method. After ID transformation, the R package “DESeq2” was used for the differential analysis between the two groups. A significance cutoff of FDR value < 0.05 and Log2 |fold change (FC)| <1 was considered as differentially expressed genes, and R package “clusterProfiler” was employed to perform GO and KEGG analysis.

### Animal studies

BALB/c nude Crlj mice (female, aged 4-5 weeks) were purchased from Viton Lever. A total number of 5 × 10^5^ U87/EGFRvIII cells were stereotactically injected into the brain of each mouse, at coordinate 3.5 mm anterior and 2.5 mm lateral of the right hemisphere relative to the bregma, at a depth of 3 mm as previously described^22^. For PDX models, a total number of 5 × 10^6^ GBM-35 and GBM-24 cells were injected. For drug treatment, mice were intraperitoneally injected with dimethyl sulfoxide (0.3%) or TMZ (60 mg/kg), DZNep (1.5 mg/kg), or TMZ/DZNep combination every other day for 2 weeks. To measure tumor growth, bioluminescence imaging was performed once a week to examine the luciferase activity. At the end of the study, all mice were humanely euthanized by carbon dioxide inhalation, followed by cervical dislocation and orthotopic tumors were collected for pathological analysis.

### Statistical analysis

Two-tailed unpaired student’s *t* test was applied to compare the variables between two groups of independent samples. To study differences between three or more groups, one-way ANOVA with LSD-t was used. For correlation analysis, *Pearson* correlation analysis was utilized. *P* < 0.05 were considered as statistically significant.

### Reporting summary

Further information on research design is available in the Nature Portfolio Reporting Summary linked to this article.

## Supplementary information

Supplementary Information

Reporting Summary

## Data Availability

All remaining data supporting the conclusions of this study are available in the article and supplementary files. The RNA sequencing data were uploaded to the Gene Expression Omnibus (GEO) public database (GSE217347). The mass spectrometry proteomics data have been deposited to the ProteomeXchange Consortium (PXD037869). Source data are provided with the paper. Source data are provided with this paper.

## Source data

Source Data
