# Supplementary material for: Lysine methylation promotes NFAT5 activation and determines temozolomide efficacy in glioblastoma
Source: Nat Commun. 2023 Jul 10;14:4062. doi: 10.1038/s41467-023-39845-z (PMC10333326; doi:10.1038/s41467-023-39845-z)
Supplement: Supplementary file 1 — Supplementary Information [file 41467_2023_39845_MOESM1_ESM.pdf]

Lysine methylation promotes NFAT5 activation and determines temozolomide efficacy in glioblastoma

Yatian Li[#,1], Zhenyue Gao[#,2], Yuhong Wang[#,1], Bo Pang[#,3], Binbin Zhang[4], Ruxin Hu[2], Yuqing Wang[2], Chao Liu[5,6], Xuebin Zhang[7], Jingxuan Yang[8,9], Mei Mei*[,2], Yongzhi Wang*[,3], Xuan Zhou*[,5,6], Min Li*[,8,9] and Yu Ren*[,1,8,9]

[1]Department of Genetics, School of Basic Medical Sciences, Tianjin Medical University, Tianjin, China; [2]Department of Cell Biology, School of Basic Medical Sciences, Tianjin Medical University, Tianjin, China; [3]Beijing Neurosurgical Institute, Beijing Tiantan Hospital, Capital Medical University, Beijing, China; [4]Department of Neuro-oncology, Tianjin Huanhu Hospital, Tianjin, China; [5]Department of Maxillofacial and Otorhinolaryngology Oncology, Tianjin Medical University Cancer Institute & Hospital, Tianjin, China; [6]Key Laboratory of Cancer Prevention and Therapy, Tianjin Cancer Institute, National Clinical Research Center of Cancer, Tianjin, China; [7]Department of Pathology, Tianjin Huanhu Hospital, Tianjin, China; [8]Department of Medicine, The University of Oklahoma Health Sciences Center, Oklahoma City, Oklahoma, USA; [9]Department of Surgery, The University of Oklahoma Health Sciences Center, Oklahoma City, Oklahoma, USA.

This PDF file includes:

Supplementary Materials

Fig. S1 to S12

Table S1 to S5

Supplementary Figures

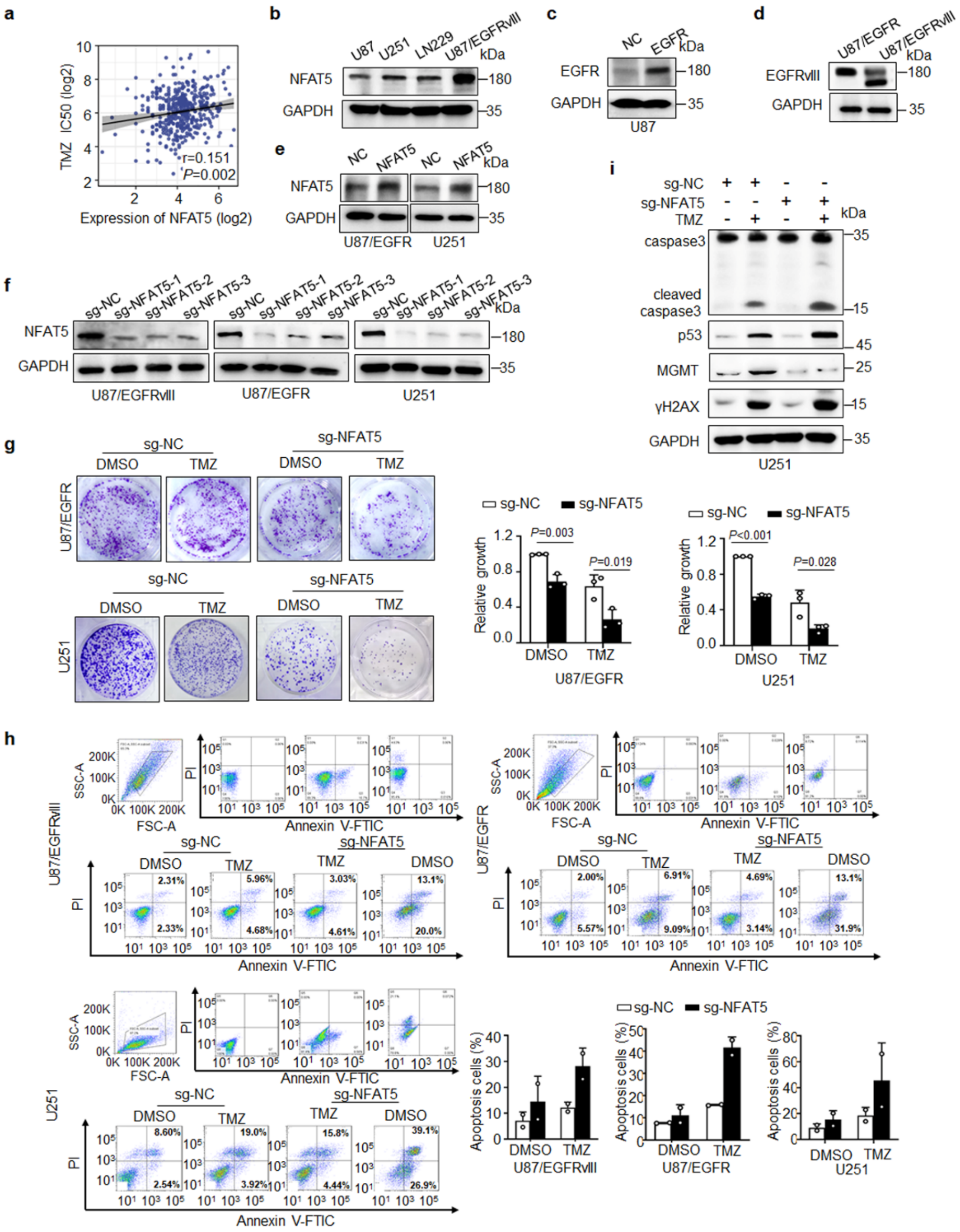

Supplementary Figure 1. NFAT5 expression correlates with the response to TMZ therapy.

a, The association between NFAT5 expression and TMZ response form 505 cancer cell lines from the Genomics of Drug Sensitivity in Cancer (GDSC) database. **b.** Western blot analysis of NFAT5 protein expression in different GBM cell lines. **c.** EGFR protein

levels in con and EGFR overexpressing stable clones of U87 cells. **d.** EGFRvIII protein levels in U87/EGFR and U87/EGFRvIII cells. **e.** The protein expression of NFAT5 in control and overexpression NFAT5 stable clones of U87/EGFR and U251 cells. **f.** The protein expression of NFAT5 in sg-NC and sg-NFAT5 stable clones of U87/EGFRvIII, U87/EGFR and U251 cells. **g,** Colony formation assay in U87/EGFR and U251 cells expressing sg-NC or sg-NFAT5 with or without TMZ treatment (200μM). **h,** Flow cytometry analysis of the effect of NFAT5 knockout on the percentage of apoptotic cells in U87/EGFRvIII, U87/EGFR and U251 cells with or without TMZ treatment. **i,** The protein levels of MGMT and cleaved caspase3 in NFAT5 overexpressing U251 cells treated with or without TMZ. (**b-g, i**) n = 3 independent experiments. (**h**) n = 2 independent experiments; Significance was calculated by (**a**) Pearson test; (**g**) unpaired Student's t test. Data was presented as mean ± standard deviation. Marker unit for Western blots is kDa. Source data are provided as a Source Data file.

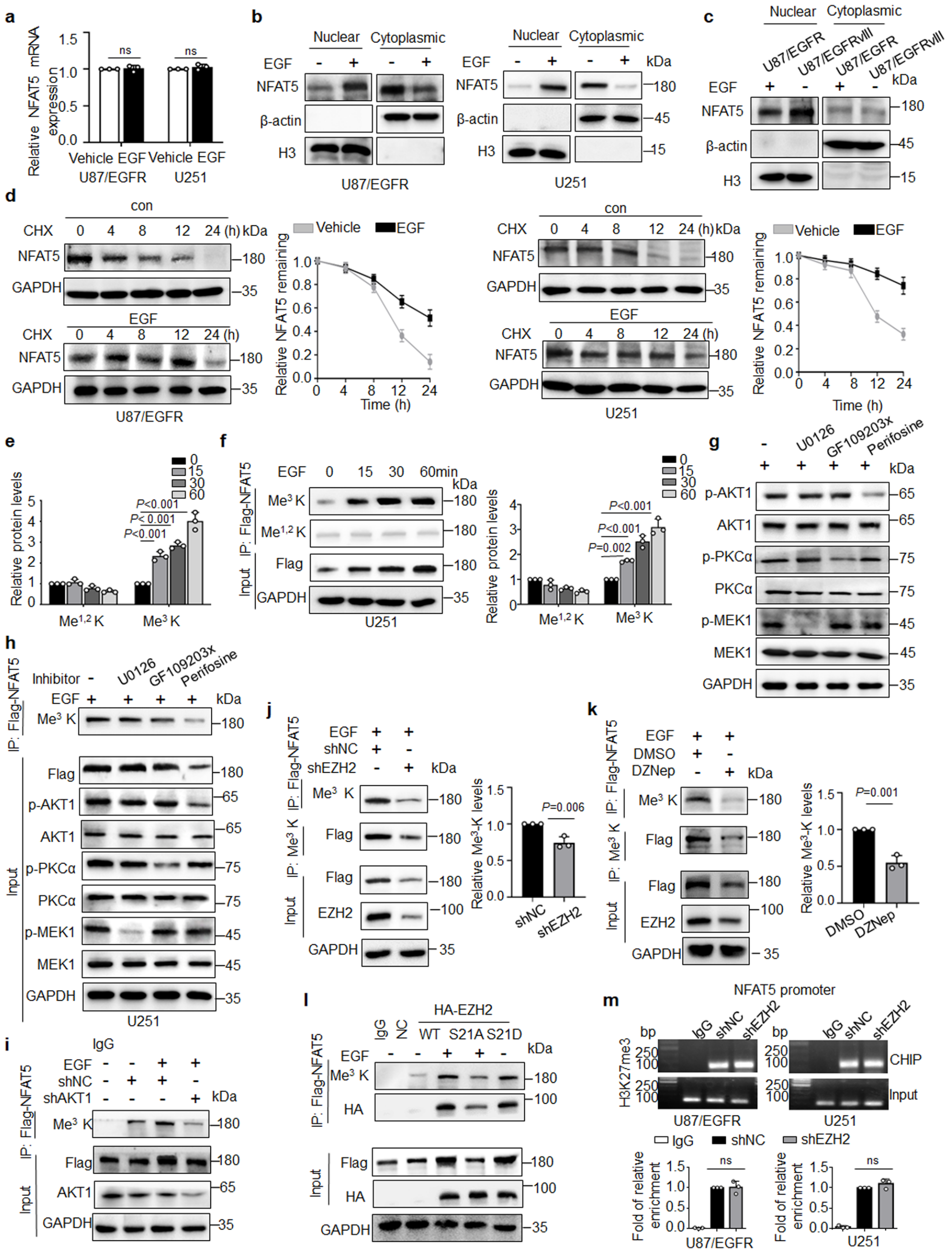

Supplementary Figure 2. EZH2 is required for EGF-induces NFAT5 upregulation and lysine methylation.

a, NFAT5 mRNA expression in U87/EGFR and U251 with or without EGF treatment for 15min. **b,** The nuclear and cytosolic NFAT5 protein levels in U87/EGFR and U251 cells treated with or without EGF. **c,** The nuclear and cytosolic NFAT5 protein levels in U87/EGFR and U87/EGFRvIII cells. **d,** Degradation of NFAT5 was assessed by CHX

treatment with or without EGF stimulation in U87/EGFR and U251 cells. Right, quantification of the NFAT5 intensity. **e,** Quantification of the Me^{3} K and $Me^{1,2}$ K levels shown in Fig. 3c. **f,** The effect of different incubation times of EGF treatment on the expression and lysine methylation levels of NFAT5 in U251 cells. Right, quantification of the Me^{3} K and $Me^{1,2}$ K levels. **g,** U87/EGFR and U251 cells (**h**) were pretreated with 10 μM PKCα (GF109203X), 10 μM MEK1 (U0126), or 10 μM AKT1 (perifosine) inhibitors for 24 h followed by EGF (100 ng/mL) for 15min. Lysates were subjected to immunoblotting analysis. **i,** Knockdown of AKT1 by shRNA reduced for EGF-induced NFAT5 tri-lysine methylation. **j,** Knockdown of EZH2 by shRNA or small molecule inhibitor, DZNep treatment (**k**) reduced EGF-induced NFAT5 lysine methylation in U251 cells. Right, quantification of the Me^{3}K intensity. **l,** NFAT5 tri-lysine methylation expression and the association between NFAT5 and EZH2 in U251 cells expressing EZH2-WT, EZH2-S21A mutant, or EZH2-S21D mutant. **m,** CHIP assay revealed that knockdown of EZH2 perform no significance of $H3K27me^{3}$ binding to the promoter of NFAT5 in U87/EGFR and U251 cells. (**a-d, f-m**) n = 3 independent experiments. Significance was calculated by (**e, f**) one way ANOVA with LSD-t; by (**j, k**) unpaired Student's t test. Data was presented as mean ± standard deviation. Marker unit for Western blots is kDa. Marker size for CHIP is bp. Source data are provided as a Source Data file.

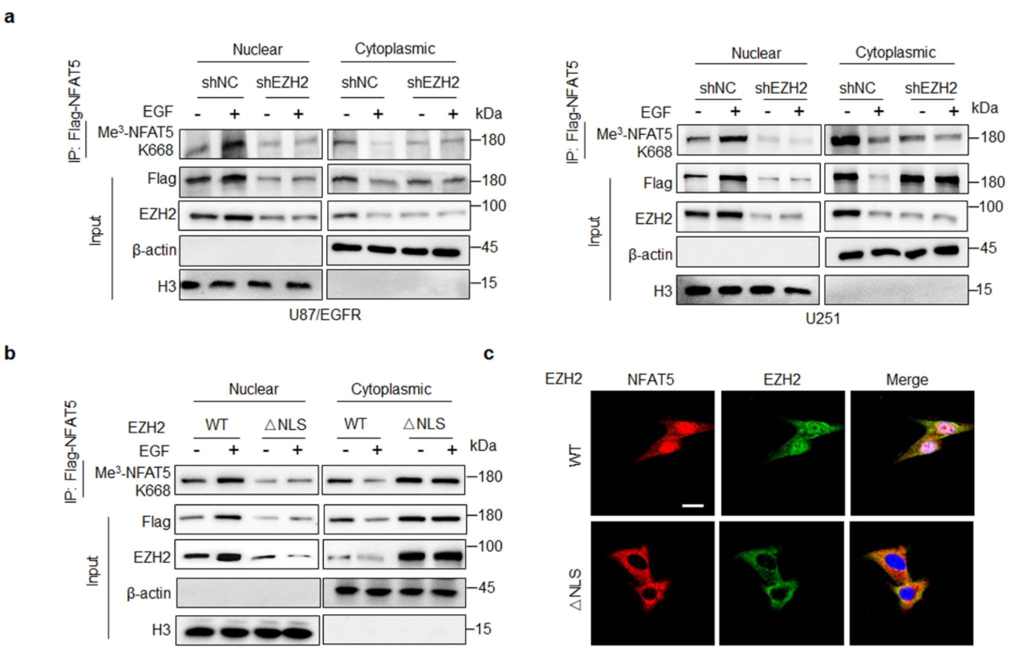

Supplementary Figure 3. EZH2 is required for EGF induced NFAT5 nuclear translocation.

a, The nuclear and cytosolic expression of NFAT5 and Me^{3}-NFAT5 K668 was detected in U87/EGFR or U251 cells transfected with shNC or shEZH2 in the presence of EGF or not. **b,** The nuclear and cytosolic protein levels of NFAT5 and Me^{3}-NFAT5 K668 was detected in U251 cells transfected with EZH2 WT or EZH2 NLS mutant in the presence of EGF or not. NLS, nuclear localization signal. **c,** Representative images of IF staining of NFAT5 in U251 cells expressing EZH2 WT or NLS mutant after incubation with EGF for 15 min. Scale bar: 20μm. One representative field of n = 30 independent cells was captured. (**a-c**) n = 3 independent experiments. Marker unit for Western blots is kDa. Source data are provided as a Source Data file.

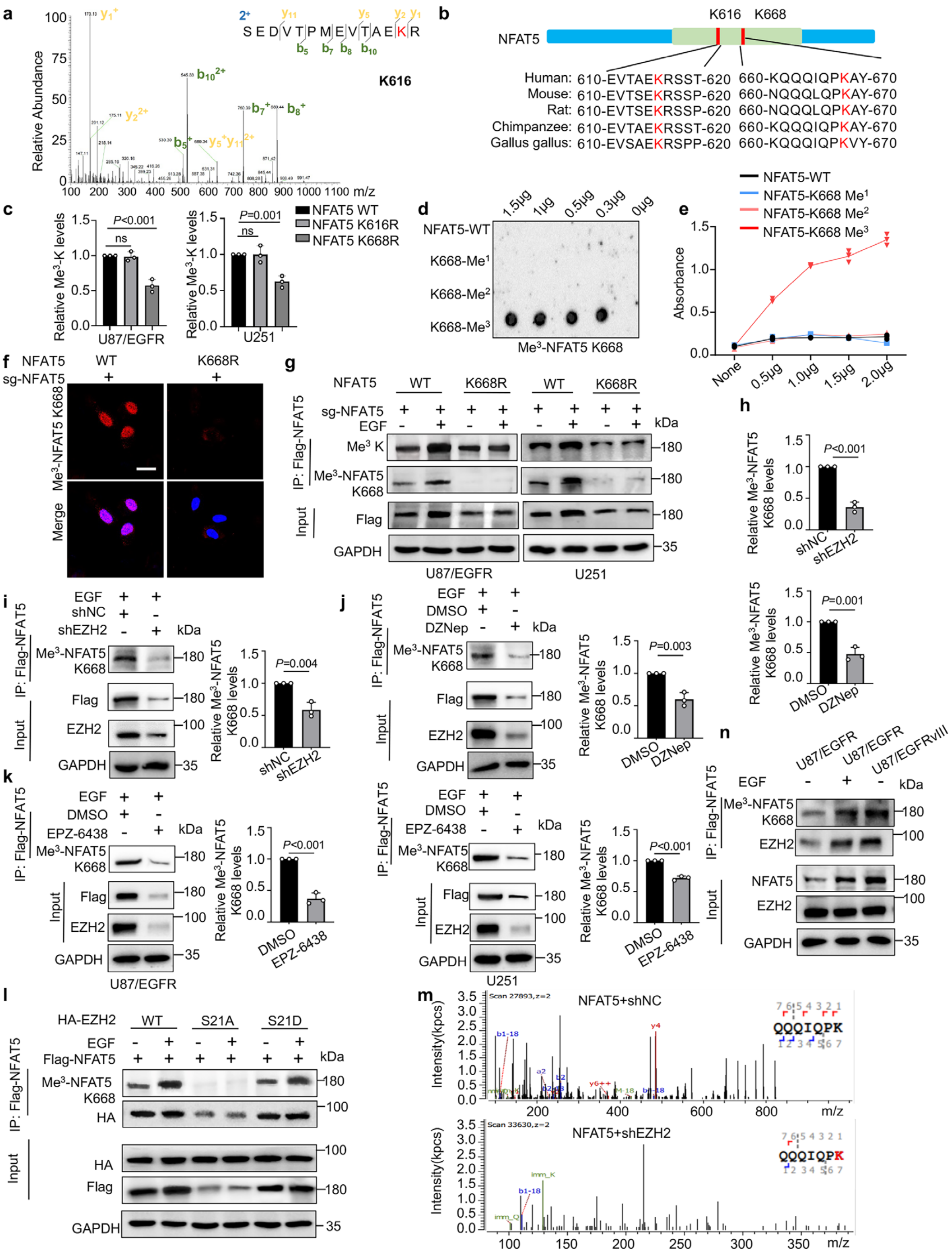

Supplementary Figure 4. EZH2 methylates NFAT5 at K668.

a, LC-MS/MS-based identification corresponding to the K616 site lysine methylation of NFAT5 (from U251 cells). **b,** The K616 and K668 amino acid sequence of NFAT5 alignment from distinct species. **c,** Quantification of the Me^3 K levels shown in Fig.4b. **d,** Dot blot and ELISA (**e**) analysis showing the Me^3-NFAT5 K668 antibody specifically recognized NFAT5-K668 Me^3 peptide. **f,** Representative images of IF

staining of Me^3-NFAT5 K668 in U251 cells expressing NFAT5 WT or K668R mutant after incubation with EGF for 15 min. Scale bar: 20μm. One representative field of *n* = 30 independent cells was captured. **g,** The expression levels of Me^3K and Me^3-NFAT5 K668 in cells expressing NFAT5 WT or K668R mutant. **h,** The protein qualification of Me^3-NFAT5 K668 levels in Fig. 4c. **i,** Knockdown of EZH2 by shRNA or DZNep treatment (**j**) mitigated EGF-induced NFAT5 K668 methylation in U251 cells. Right, quantification of the Me^3-NFAT5 K668 intensity. **k,** EPZ-6438 treatment reduced Me^3-NFAT5 K668 expression in U87/EGFR and U251 cells. Right, quantification of the Me^3-NFAT5 K668 intensity. **l,** Expression of Me^3-NFAT5 K668 and the association between NFAT5 and EZH2 was analyzed in HA-tagged EZH2 WT, S21A, S21D expressing U87/EGFR cells. **m,** Stoichiometry of NFAT5 K668 methylation by EZH2 based on liquid chromatography tandem mass spectrometry. **n,** Western blotting was performed to examine the Me^3-NFAT5 K668 in U87/EGFRvIII cells and U87/EGFR cells with or without EGF treatment. (**d-g, i-l, n**) *n* = 3 independent experiments. Significance was calculated by (**c**) one way ANOVA with LSD-t; by (**h-k**) unpaired Student's t test. Data was presented as mean ± standard deviation. Marker unit for Western blots is kDa. Source data are provided as a Source Data file.

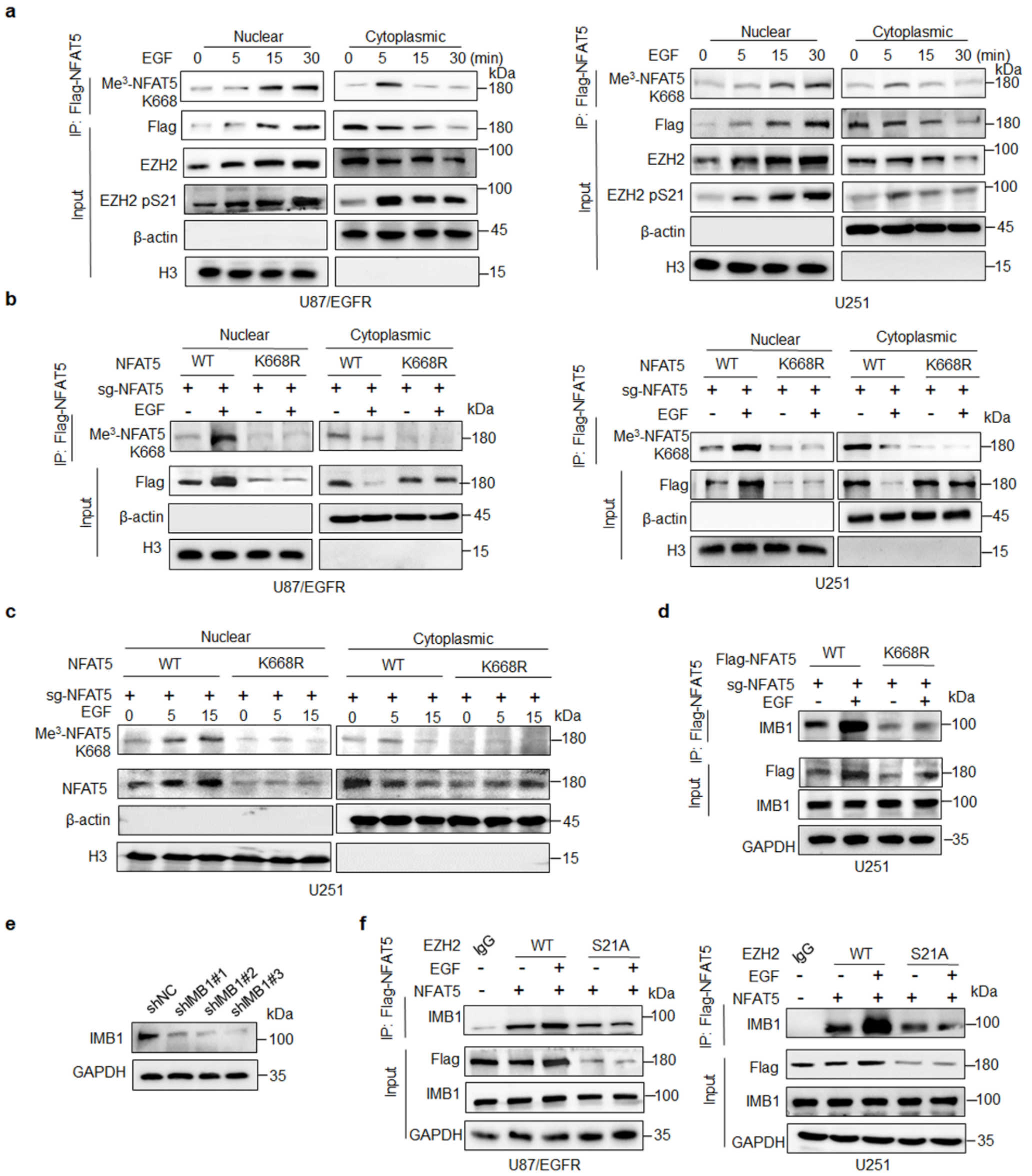

Supplementary Figure 5. Methylation of NFAT5 at K668 is crucial for NFAT5 binding to IMB1 and nuclear localization.

a, The nuclear and cytosolic fractions of U87/EGFR and U251 cells treated with or without EGF were collected for IP with Flag antibody, followed by IB analysis (n=2 independent experiments). **b,** The nuclear and cytosolic fractions of U87/EGFR and U251 cells expressing NFAT5 WT or K668R mutant treated with or without EGF were subjected to immunoblotting analysis. **c,** The nuclear and cytosolic fractions of U251 cells expressing NFAT5 WT or K668R mutant treated with EGF at different time were

subjected to immunoblotting analysis. **d,** The association of NFAT5 and IMB1 was examined in NFAT5 WT or K668R U251 cells in the presence of EGF or not. **e,** The protein expression of IMB1 in shNC and shIMB1 stable clones of U251 cells. **f,** The interaction of NFAT5 and IMB1 was analyzed in cells transfected with EZH2 WT or S21A mutant. (**a-f**) $n = 3$ independent experiments. Marker unit for Western blots is kDa. Source data are provided as a Source Data file.

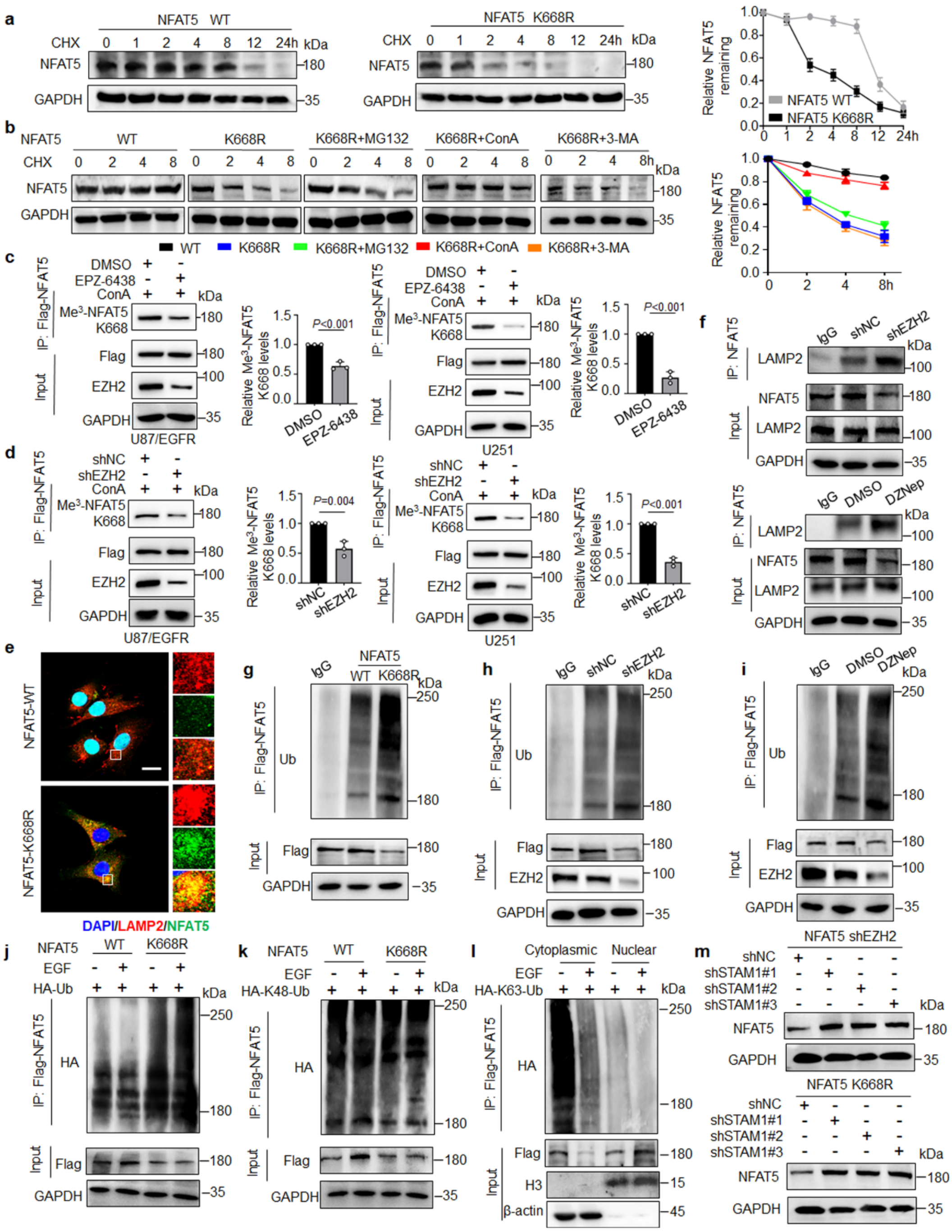

Supplementary Figure 6. NFAT5 K668 methylation inhibits K63-linked ubiquitination of NFAT5 lysosome degradation.

a, Degradation of NFAT5 was assessed by CHX treatment in NFAT5 WT or K668R mutant expressing U251 cells. Right, quantification of the NFAT5 intensity. **b,** Degradation of NFAT5 was assessed by CHX treatment in the presence of proteasome

inhibitor (MG132), inhibitor of autophagy (3-MA) or lysosome (ConA) in NFAT5 WT or K668R mutant expressing cells. Right, quantification of the NFAT5 intensity. **c,** Knockdown of EZH2 by EPZ-6438 treatment or shRNA (**d**) in the presence of concanamycin-A treatment reduced Me^3-NFAT5 K668 levels in U87/EGFR and U251 cells, without changing the total levels of NFAT5. Right, quantification of the Me^3-NFAT5 K668 intensity. **e,** Representative images of IF staining of the colocalization of NFAT5 with the lysosome marker LAMP2 in U251 cells expressing NFAT5 WT or K668R. Scale bar, 20μm. One representative field of n = 15 independent cells was captured. **f,** Knockdown of EZH2 by shRNA or DZNep markedly induced the interaction of NFAT5 and LAMP2. **g,** NFAT5 K668R mutant markedly increased NFAT5 ubiquitination compared to NFAT5-WT cells. **h,** Knockdown of EZH2 by shRNA or DZNep (**i**) markedly enhanced NFAT5 ubiquitination. **j,** EGF reduced NFAT5 ubiquitination in NFAT5 WT but not K668R mutant expressing cells. **k,** No significant difference was found in EGF induced NFAT5 K48-linked ubiquitination. **l,** Immunoprecipitation assay was performed for NFAT5 K63-linked ubiquitin in the cytosol and nuclear fraction of U251 cells. **m,** STAM1 deficiency restored NFAT5 expression in shEZH2 transfection or NFAT5 K668R expressing U251 cells. (**a-m**) n = 3 independent experiments. Significance was calculated by (**c and d**) unpaired Student's t-test. Marker unit for Western blots is kDa. Source data are provided as a Source Data file.

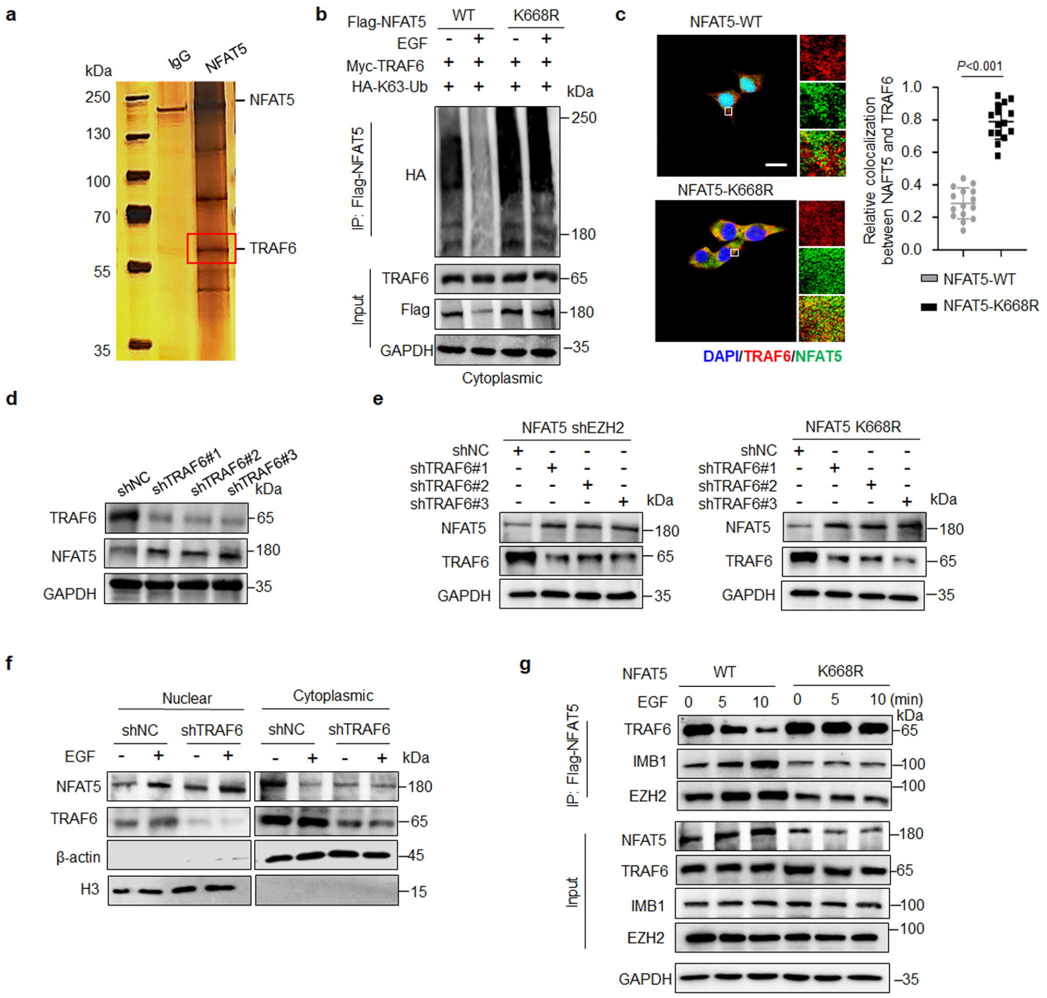

Supplementary Figure 7. Methylation stabilizes NFAT5 and facilitates its nuclear abundance by blocking its association with E3 ligase TRAF6.

a, Mass-spectrometry identified the potential E3-ligase that bound to the NFAT5. **b,** TRAF6 induced cytoplasmic NFAT5 K63-linked ubiquitination in NFAT5 WT but not K668R expressing cells. **c,** Representative images of IF staining of the colocalization of NFAT5 with TRAF6 in U251 cells expressing NFAT5 WT or K668R. Scale bar, 20μm. *n* =15 independent cells. **d,** The protein expression of NFAT5 in shNC or shTRAF6 cells. **e,** TRAF6 deficiency restored NFAT5 expression in shEZH2 transfection or NFAT5 K668R expressing cells. **f,** The nuclear and cytosolic fractions of U251 cells transfected with shNC and shTRAF6 treated with or without EGF were

subjected to immunoblotting analysis. **g,** U251 cells were treated with EGF at indicated time, and whole-cell extracts were collected for IP with Flag antibody, followed by IB analysis. (**b-g**) $n = 3$ independent experiments. Significance was calculated by (**c**) unpaired Student's t-test. Data was presented as mean ± standard deviation. Marker unit for Western blots is kDa. Source data are provided as a Source Data file.

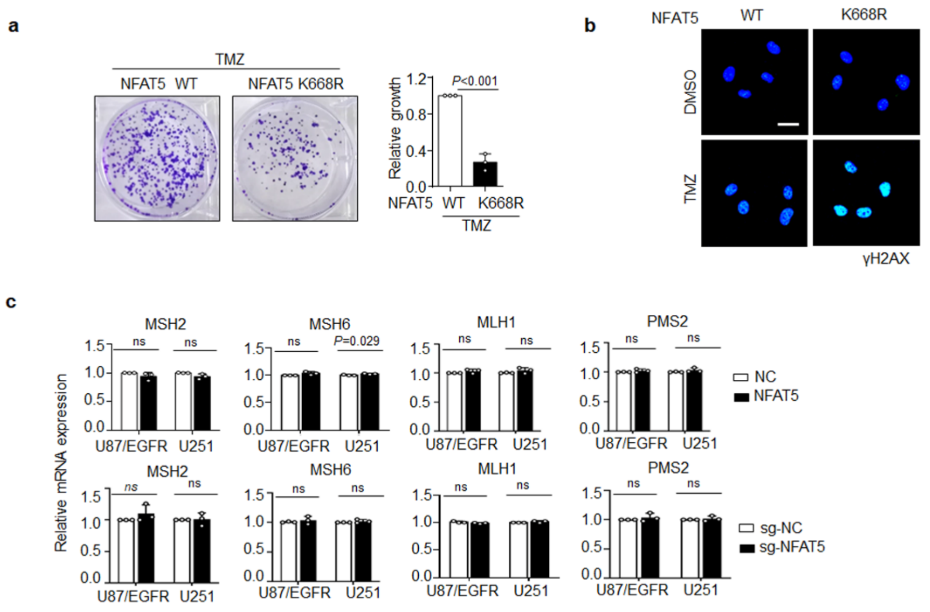

Supplementary Figure 8. NFAT5 K668 methylation regulates TMZ efficacy.

a, Colony formation assay in U87/EGFRvIII cells expressing NFAT5 WT or K668R with TMZ treatment. **b**, Representative images of IF staining of γH2AX in NFAT5 WT or K668R mutant-transfected U87/EGFR cells treated with TMZ therapy. Scale bar: 20μm. One representative field of n = 30 independent cells was captured. **c**, The effect of NFAT5 on mRNA levels of MMR related genes in U87/EGFR cells and U251 cells. (**a-c**) n = 3 independent experiments; Significance was calculated by (**a, c**) unpaired Student's t-test. Data was presented as mean ± standard deviation. Source data are provided as a Source Data file.

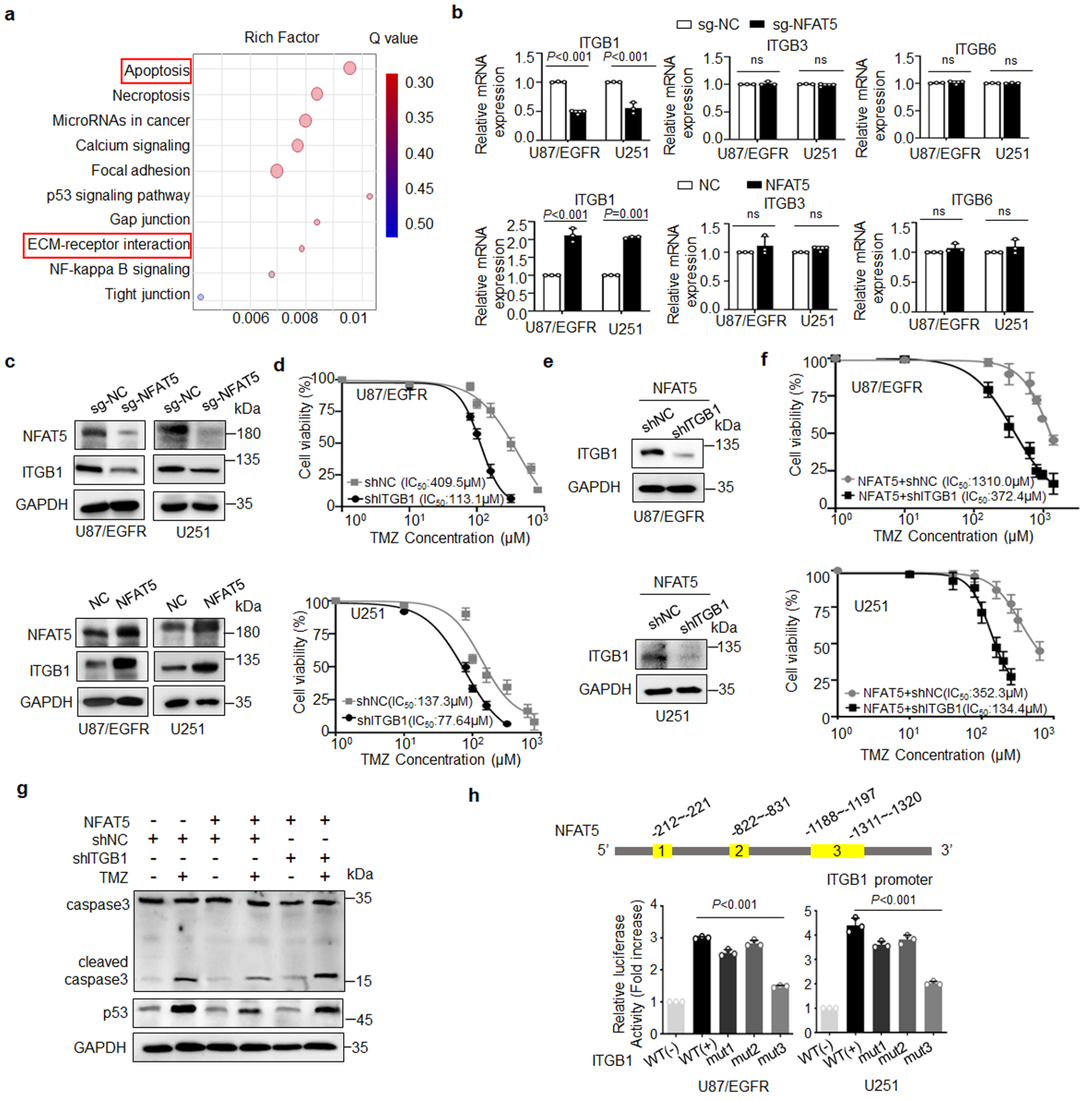

Supplementary Figure 9. ITGB1 is required for NFAT5 induced poor TMZ efficacy.

a, Kyoto Encyclopedia of Genes and Genomes (KEGG) pathway enrichment analysis revealed that the differentially expressed genes between the NFAT5-overexpressing and control cells were enriched in the following pathways: “Apoptosis” and “ECM-receptor interaction.” **b,** The effect of NFAT5 on ITGB1, ITGB3 and ITGB6 mRNA expression in U87/EGFR and U251 cells. **c,** The effect of NFAT5 on ITGB1 protein expression in U87/EGFR and U251 cells. **d,** CCK-8 assay analysis revealed the effect of shITGB1 on TMZ treatment efficacy at the indicated concentrations for 72 h in U87/EGFR and U251

cells. **e,** The protein expression of ITGB1 in U87/EGFR and U251 overexpressing NFAT5 cells transfected with shNC and shITGB1. **f,** CCK-8 assay analysis revealed the effect of shITGB1 on TMZ treatment efficacy at the indicated concentrations for 72 h in U87/EGFR and U251 overexpressing NFAT5 cells. **g,** The effect of ITGB1 on cleaved caspase3 expression in U251 cells incubated with or without TMZ transfected with or without NFAT5. **h,** The results of the dual-luciferase reporter assay revealed that ITGB1 was a direct transcriptional target of NFAT5. (**b-h**) n = 3 independent experiments; Significance was calculated by (**b, h**) unpaired Student's t-test. Data was presented as mean ± standard deviation. Marker unit for Western blots is kDa. Source data are provided as a Source Data file.

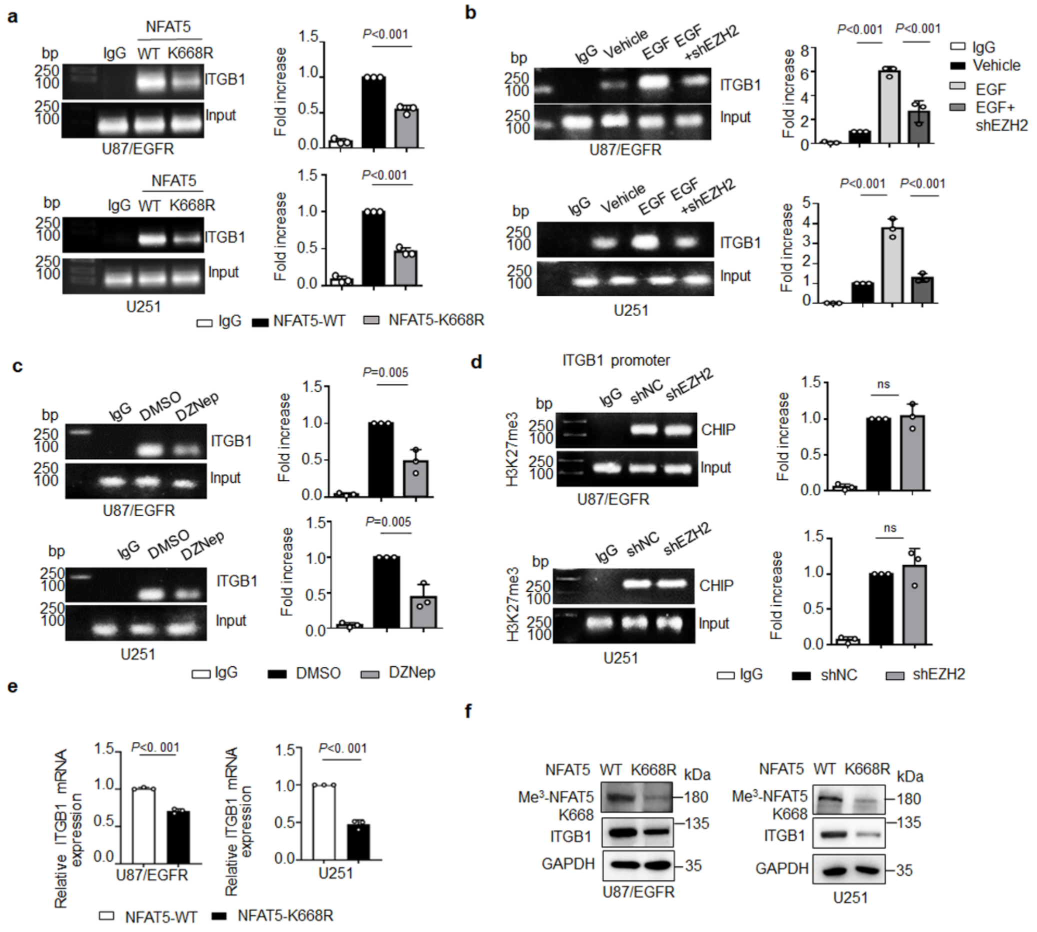

Supplementary Figure 10. K668 methylation is required for NFAT5-induced *ITGB1* transcription.

a, ChIP assays shown that NFAT5 K668R mutation reduced NFAT5 binding affinity to *ITGB1* promoter, compare to the NFAT5 WT cells. **b**, The results of the CHIP assay revealed that EZH2 knockdown by shRNA or DZNep treatment (**c**) decreased the binding affinity of NFAT5 to the promoter of ITGB1 in U87/EGFR and U251 cells. **d,** CHIP assay revealed that knockdown of EZH2 perform no significance of H3K27me^{3} binding to the promoter of ITGB1 in U87/EGFR and U251 cells. **e**, The mRNA and protein levels (**f**) of ITGB1 in U87/EGFR and U251 cells expressing NFAT5 WT or K668R mutation. (**a-f**) $n = 3$ independent experiments. Significance was calculated by (**a-e**) unpaired Student's t-test. Data was presented as mean ± standard deviation.

Marker unit for Western blots is kDa. Marker size for CHIP is bp. Source data are provided as a Source Data file.

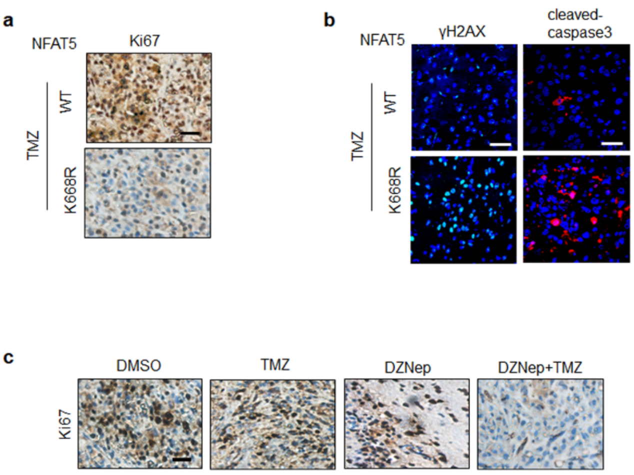

Supplementary Figure 11. Inhibition of NFAT5 K668 methylation improves TMZ efficacy in vivo.

a, IHC staining of Ki67 in tumors derived from NFAT5 WT or K668R mutant-transfected U87/EGFRvIII cells treated with TMZ therapy. Scale bar: 100µm. **b,** IF staining of γH2AX and cleaved-caspase3 in tumors shown in (a). n = 5 randomly captured field of view. Scale bar: 50µm. **c,** IHC staining of Ki67 in tumors derived from U87/EGFRvIII cells treated with TMZ, DZNep alone or in combination therapy. Scale bar: 100µm. (**a-c**) n = 2 independent experiments.

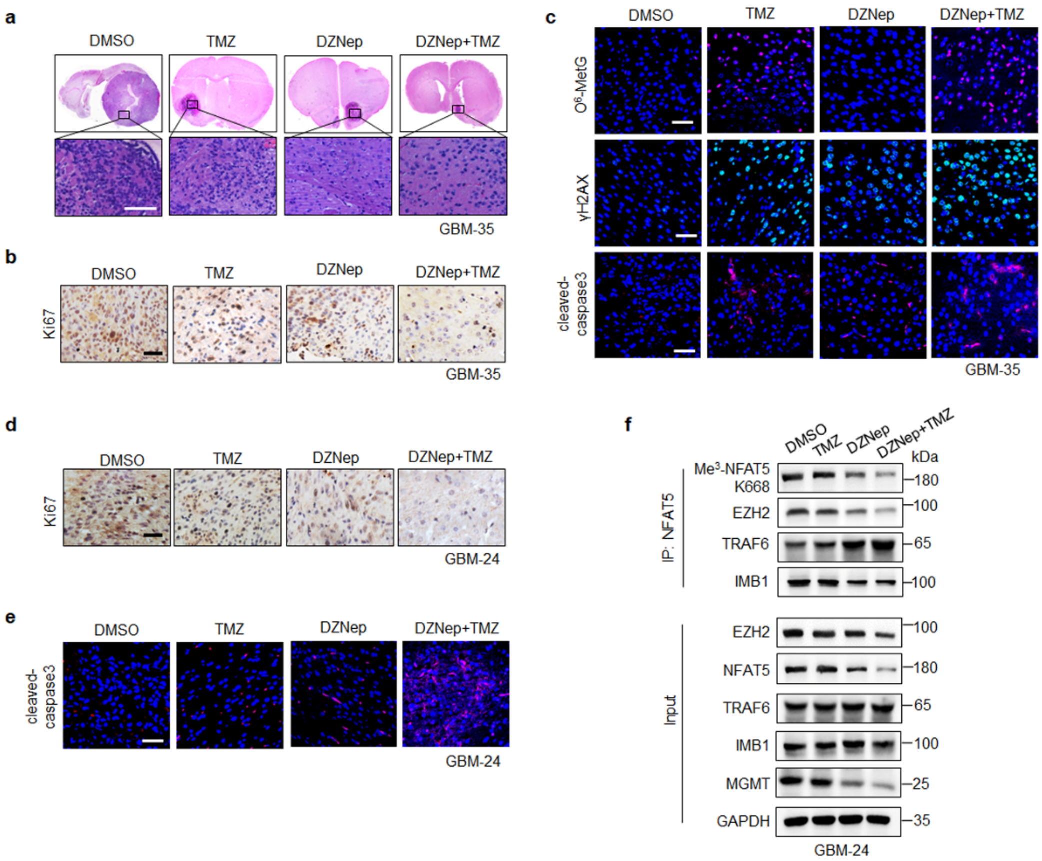

Supplementary Figure 12. Inhibition of NFAT5 K668 methylation improves TMZ efficacy in PDX models.

a, H&E-stained coronal brain sections of mice harboring GBM-35 tumors treated with TMZ, DZNep, alone or in combination. Scale bar: 100μm. **b,** IHC staining of Ki67 in the tumors from experiments shown in (a). Scale bar: 100μm. **c**, IF staining of O^6-MetG, γH2AX and cleaved-caspase3 in the tumors from experiments shown in (a). n = 5 randomly captured field of view. Scale bar: 50μm. **d,** IHC staining of Ki67 in the coronal brain sections of mice harboring GBM-24 tumors treated with TMZ, DZNep, alone or in combination. Scale bar: 100μm. **e**, IF staining of cleaved-caspase3 expression in the tumors from experiments shown in (d). n = 5 randomly captured field of view. Scale bar: 50μm. **f**, The expression levels of Me^3-NFAT5-K668, MGMT and the interaction between NFAT5 and EZH2 as well as IMB1 in the tumors from

experiments shown in (d). (**a-f**) $n = 2$ independent experiments. Marker unit for Western blots is kDa. Source data are provided as a Source Data file.

Supplementary Table

Table S1. Clinico-pathological characteristics of 83 glioma patients

**Variant**	**Total**	**NFAT5 expression**		χ^2	***P* Value**
		**Low/Medium (n=34)**	**High (n=49)**		
**Sex**					
Male	52	22	30	0.1040	0.7471
Female	31	12	19		
**Age**					
<50	41	19	22	0.9689	0.3250
≥50	42	15	27		
**Tumor location**					
Frontal	33	16	17	1.2813	0.2577
Non-frontal	50	18	32		
**KPS score**					
≥80	58	25	33	4.7661	**0.0290**
< 80	25	9	16		
**Ki-67**					
Wild	41	23	18	7.6733	**0.0056**
Mutant	42	11	31		
**MGMT promotor**					
Methylated	38	23	15	11.0912	**0.0009**
Unmethylated	45	11	34		
**Extent of surgery**					
Total	52	19	33	1.1275	0.2883
Subtotal	31	15	16		

Abbreviations: KPS, Karnofsky performance status; MGMT, O-6-methylguanine-DNA-methyltransferase

Table S2. Clinico-pathological characteristics of 55 GBM patients

Variant	Total	NFAT5 expression		χ^2	*P* Value
		Low/Medium (n=17)	High (n=38)		
**Sex**					
Male	33	10	23	0.0142	0.9052
Female	22	7	15		
**Age**					
<50	21	7	14	0.0935	0.7598
≥50	34	10	24		
**Tumor location**					
Frontal	25	12	13	6.2691	**0.0123**
Non-frontal	30	5	25		
**KPS score**					
≥80	38	11	27	0.2215	0.6379
< 80	17	6	11		
**MGMT promotor**					
Methylated	26	14	12	12.1481	**0.0005**
Unmethylated	29	3	26		
**Extent of surgery**					
Total	30	7	23	1.7737	0.1829
Subtotal	25	10	15		
**Subtype**					
Proneural	1	0	1	0.6250	0.7320
Mesenchymal	37	11	26		
Classical	17	6	11		

Table S3. Summary of TMZ treated GBM patients

Annotation	Age	Gender	IDH1/2 genotype	MGMT promotor status	Tumor location	TMZ Response
**GBM-1**	64	Male	Wild-type	Unmethylated	Frontal	PR
**GBM-2**	30	Male	Mutation	Unmethylated	Frontal	CR
**GBM-3**	27	Female	Mutation	Unmethylated	Non-frontal	PR
**GBM-4**	47	Female	Mutation	Unmethylated	Non-frontal	PR
**GBM-5**	61	Male	Wild-type	Unmethylated	Non-frontal	PR
**GBM-6**	64	Male	Wild-type	Unmethylated	Non-frontal	PR
**GBM-7**	59	Female	Wild-type	Methylated	Frontal	PD
**GBM-8**	82	Female	Wild-type	Methylated	Non-frontal	SD
**GBM-9**	41	Female	Mutation	Methylated	Non-frontal	PD
**GBM-10**	40	Female	Mutation	Methylated	Non-frontal	PD
**GBM-11**	61	Male	Wild-type	Methylated	Frontal	PD
**GBM-12**	68	Male	Wild-type	Methylated	Frontal	SD

Abbreviations: Complete remission (CR), Partial remission (PR), progression disease (PD), Stable disease (SD)

Table S4. Primary antibodies used for Western Blotting

Material	Source	Catalog number	Dilution
NFAT5	Abcam	ab3446	1:1000
EGFR	Abcam	ab30	1:1000
AKT1(phospho S473)	Abcam	ab81283	1:1000
Phospho-EZH2(Ser21)	Abcam	ab84989	1:1000
ITGB1	Abcam	ab183666	1:5000
PKCα	Abcam	ab32376	1:1000
Phospho-PKCα(Ser657)	Abcam	ab180848	1:1000
TRAF6	Abcam	ab33915	1:1000
Ub	Abcam	ab134953	1:1000
K63-Ub	Abcam	ab179434	1:1000
$Me^{1,2}$ K	Abcam	ab23366	1:1000
LAMP2	Abcam	ab125068	1:2000
MGMT	Abcam	ab108630	1:1000
γH2AX	Abcam	ab81299	1:5000
Phospho-EGFR(Y1068)	Cell Signaling Technology	3777S	1:1000
AKT1	Cell Signaling Technology	2938S	1:1000
EZH2	Cell Signaling Technology	3147S	1:1000
Cleaved caspase3	Cell Signaling Technology	9661S	1:1000
caspase3	Cell Signaling Technology	9662S	1:1000
p53	Cell Signaling Technology	2524S	1:1000
Phospho-MEK1/2(Ser217/221)	Cell Signaling Technology	9154S	1:1000
β-actin	Cell Signaling Technology	4970S	1:1000
EGFRvIII	Cell Signaling Technology	64952S	1:1000
Myc-Tag	Cell Signaling Technology	2276S	1:1000
HRP-linked anti mouse	Cell Signaling Technology	7076S	1:10000
HRP-linked anti rabbit	Cell Signaling Technology	7074S	1:10000
Me^{3} K	PTM Biolabs	PTM-601	1:1000
HA	PTM Biolabs	PTM-5389	1:1000
GAPDH	Proteintech	60004-1-Ig	1:10000
Histone-H3	Proteintech	17168-1-AP	1:1000

Table S5. Primary antibodies used for IHC staining

Material (For IHC staining)	Source	Catalog number	Dilution
NFAT5	Abcam	ab3446	1:200
Phospho EZH2(Ser21)	Abcam	ab84989	1:200
Ki67	Abcam	ab15580	1:200
MGMT	Abcam	ab108630	1:800
Phospho-EGFR(Y1068)	Cell Signaling Technology	3777S	1:100
EZH2	Cell Signaling Technology	3147S	1:200
Cleaved caspase3	Cell Signaling Technology	9661S	1:200
