# Supplementary material for: Lysine methylation promotes NFAT5 activation and determines temozolomide efficacy in glioblastoma
Source: Nat Commun. 2023 Jul 10;14:4062. doi: 10.1038/s41467-023-39845-z (PMC10333326; doi:10.1038/s41467-023-39845-z)
Supplement: Supplementary file 2 — Reporting Summary [file 41467_2023_39845_MOESM2_ESM.pdf]

nature portfolio

Corresponding author(s): DBPR NCOMMS-21-38404B-Z

Last updated by author(s): Jun 1, 2023

Reporting Summary

Nature Portfolio wishes to improve the reproducibility of the work that we publish. This form provides structure for consistency and transparency in reporting. For further information on Nature Portfolio policies, see our Editorial Policies and the Editorial Policy Checklist.

Statistics

For all statistical analyses, confirm that the following items are present in the figure legend, table legend, main text, or Methods section.

n/a	Confirmed	
☐	☒	The exact sample size (*n*) for each experimental group/condition, given as a discrete number and unit of measurement
☐	☒	A statement on whether measurements were taken from distinct samples or whether the same sample was measured repeatedly
☐	☒	The statistical test(s) used AND whether they are one- or two-sided<br>*Only common tests should be described solely by name; describe more complex techniques in the Methods section.*
☐	☒	A description of all covariates tested
☐	☒	A description of any assumptions or corrections, such as tests of normality and adjustment for multiple comparisons
☐	☒	A full description of the statistical parameters including central tendency (e.g. means) or other basic estimates (e.g. regression coefficient) AND variation (e.g. standard deviation) or associated estimates of uncertainty (e.g. confidence intervals)
☐	☒	For null hypothesis testing, the test statistic (e.g. *F*, *t*, *r*) with confidence intervals, effect sizes, degrees of freedom and *P* value noted<br>*Give P values as exact values whenever suitable.*
☒	☐	For Bayesian analysis, information on the choice of priors and Markov chain Monte Carlo settings
☒	☐	For hierarchical and complex designs, identification of the appropriate level for tests and full reporting of outcomes
☐	☒	Estimates of effect sizes (e.g. Cohen's *d*, Pearson's *r*), indicating how they were calculated

Our web collection on statistics for biologists contains articles on many of the points above.

Software and code

Policy information about availability of computer code

Data collection	ibm SPSS statistics 27.0; FlowJo 7.6.1; Image J 1.8.0; Olympus Fluoview Ver.4.2a
Data analysis	Two-tailed unpaired student's t-test was applied to compare the variables between two groups of independent samples. To study differences between three or more groups, one-way ANOVA with LSD-t was used. For correlation analysis, Pearson correlation analysis was utilized. P < 0.05 were considered as statistically significant.

For manuscripts utilizing custom algorithms or software that are central to the research but not yet described in published literature, software must be made available to editors and reviewers. We strongly encourage code deposition in a community repository (e.g. GitHub). See the Nature Portfolio guidelines for submitting code & software for further information.

Data

Policy information about availability of data

All manuscripts must include a data availability statement. This statement should provide the following information, where applicable:

- Accession codes, unique identifiers, or web links for publicly available datasets
- A description of any restrictions on data availability
- For clinical datasets or third party data, please ensure that the statement adheres to our policy

The RNA sequencing data generated in this study have been deposited in the Gene Expression Omnibus (GEO) database under accession code (GSE217347). The mass spectrometry proteomics data have been deposited to the ProteomeXchange Consortium (PXD037869). All the data supporting the findings of this study are available within the article and its supplementary information files and from the corresponding author upon reasonable request. The source data underlying Figures 1-9 and Supplementary Figures S1-S12 are provided as a Source Data file.

Field-specific reporting

Please select the one below that is the best fit for your research. If you are not sure, read the appropriate sections before making your selection.

☒ Life sciences ☐ Behavioural & social sciences ☐ Ecological, evolutionary & environmental sciences

For a reference copy of the document with all sections, see nature.com/documents/nr-reporting-summary-flat.pdf

Life sciences study design

All studies must disclose on these points even when the disclosure is negative.

Sample size	No statistical methods were used to pre-determine sample size. The sample size of each experiment is provided in the figure/table legends in the main manuscript and supplementary file. For RNA-sequencing, samples were prepared at least in two biological replicates. For in vivo mouse model, each group has at least 4 mice. These sizes have previously been shown as sufficiently powered to determine statistical differences in mean values of our investigated.
Data exclusions	No data have been excluded from the analysis.
Replication	The replication number is indicated in the legend of corresponding figures where applicable. All attempts at replication were successful.
Randomization	Mice experimentations: For the in vivo experiments, mice were previously randomized into experimental groups, because all mice were the same age and sex and were purchased from the same supplier. Cell line experimentation: For each experiment, the total amount of cells from one cell line required for all tested conditions were pooled and seeded randomly into different plates, pre-labeled with the treatment to be applied.
Blinding	All the control and experimental group of mice/cells were grown under identical conditions. No blinding were used.

Reporting for specific materials, systems and methods

We require information from authors about some types of materials, experimental systems and methods used in many studies. Here, indicate whether each material, system or method listed is relevant to your study. If you are not sure if a list item applies to your research, read the appropriate section before selecting a response.

Materials & experimental systems

n/a	Involved in the study	
☐	☒	Antibodies
☐	☒	Eukaryotic cell lines
☒	☐	Palaeontology and archaeology
☐	☒	Animals and other organisms
☐	☒	Human research participants
☒	☐	Clinical data
☒	☐	Dual use research of concern

Methods

n/a	Involved in the study	
☒	☐	ChIP-seq
☐	☒	Flow cytometry
☒	☐	MRI-based neuroimaging

Antibodies

Antibodies used	anti-NFAT5 (ab3446), anti-EGFR (ab30), anti-AKT1 (phospho S473) (ab81283), anti-EZH2 S21 (ab84989), anti-MGMT (ab108630), anti-ITGB1 (ab183666), anti-PKCα (ab32376), anti-phospho-PKCα S657 (ab180848), anti-TRAF6 (ab33915), anti-Ub (ab134953) ,anti-K63-Ub (ab179434), anti-Me1,2 K (ab23366) ,anti-LAMP2(ab125068) and anti-γH2AX (ab81299) were purchased from Abcam. Anti-phospho-EGFR Y1068 (3777S), anti-AKT1 (2938S), anti-EZH2 (3147S), anti-cleaved caspase3 (9661S), anti-caspase3 (9662S), anti-p53 (2524S), Phospho-MEK1/2(Ser217/221) (9154S), anti-β-actin (4970S), anti-EGFRvIII (64952S), anti-Myc-Tag (2276S), HRP-linked anti-mouse (7076S) and anti-rabbit (7074S) were purchased from Cell Signaling Technology. Anti-GAPDH (60004-1-Ig) and anti-histone-H3 (17168-1-AP) were purchased from proteintech. All primary antibodies were used in a dilution of 1:1000, expect anti-ITGB1 and anti-γH2AX used in 1:5000 ,anti-LAMP2 used in 1:2000 and anti-GAPDH used in 1:10000, and the secondary antibodies were used in 1:10000 dilution.
Validation	All commercially available antibodies were validated by vendors. Validation statements are provided on the manufacture's website. We examined primary antibodies according to manuals, and got similar results with validation results on manufacturer's website or relevant citations.

Eukaryotic cell lines

Policy information about cell lines

Cell line source(s)	Human GBM cell lines (U87and LN229 cells) were purchased from the American Type Culture Collection (ATCC). U251 cells were purchased from Millipore.The U87/EGFRvIII cell line was a kind gift from Dr. Lei Han (Tianjin Medical University General Hospital, Tianjin, China).GBM-35 and GBM-24, these two cell lines were derived from two patients with GBM, which were obtained from the Beijing Institute of Neurosurgery, Beijing Tiantan Hospital.
Authentication	Cell lines were obtained from original sources and were not further authenticated
Mycoplasma contamination	All cell lines used in this study were negative for mycoplasma.
Commonly misidentified lines (See ICLAC register)	No commonly misidentified cell lines were used in the study.

Animals and other organisms

Policy information about studies involving animals; ARRIVE guidelines recommended for reporting animal research

Laboratory animals	BALB/c nude Crlj mice (female, aged 4-5 weeks) were purchased from Viton Lever.
Wild animals	This study did not involve wild animals.
Field-collected samples	None
Ethics oversight	All animal experiments were approved by the Ethics Committee of the Tianjin Medical University (TMUaMEC2017016).

Note that full information on the approval of the study protocol must also be provided in the manuscript.

Human research participants

Policy information about studies involving human research participants

Population characteristics	Adult patients with GBM.
Recruitment	Participants were recruited from the pool of patients at Tianjin Huanhu Hospital. No selection bias was observed
Ethics oversight	All patients voluntarily signed an informed consent form. This study was approved by the Tianjin Huanhu Hospital ethical committee (EK 2019179).

Note that full information on the approval of the study protocol must also be provided in the manuscript.

Flow Cytometry

Plots

Confirm that:

☒ The axis labels state the marker and fluorochrome used (e.g. CD4-FITC).

☒ The axis scales are clearly visible. Include numbers along axes only for bottom left plot of group (a 'group' is an analysis of identical markers).

☒ All plots are contour plots with outliers or pseudocolor plots.

☒ A numerical value for number of cells or percentage (with statistics) is provided.

Methodology

Sample preparation	Apoptosis was determined using flow cytometry with the Annexin V-FITC/PI Apoptosis detection kit (Elabscience, E-CK-A211).
Instrument	FACSVerse (BD Biosciences)
Software	FlowJo 7.6.1 was used for analysis.
Cell population abundance	Between 10,000 and 50,000 cells were acquired per sample, and the total population was analysed.
Gating strategy	Cellular apoptosis was measured using the Annexin V-FITC/PI kit according to the manufacturer's protocol. FITC and PI fluorescence were both excited at 488 nm, while they were collected at 525 nm and 630 nm, respectively.

☒ Tick this box to confirm that a figure exemplifying the gating strategy is provided in the Supplementary Information.
